# Nociceptor sodium channels shape subthreshold phase, upstroke, and shoulder of action potentials

**DOI:** 10.1085/jgp.202313526

**Published:** 2025-01-21

**Authors:** Phil Alexander Köster, Enrico Leipold, Jenny Tigerholm, Anna Maxion, Barbara Namer, Thomas Stiehl, Angelika Lampert

**Affiliations:** 1 Institute for Neurophysiology, Uniklinik RWTH Aachen University, Aachen, Germany; 2 Scientific Center for Neuropathic Pain Aachen SCN^AACHEN^, Uniklinik RWTH Aachen University, Aachen, Germany; 3 Department of Anesthesiology and Intensive Care and CBBM-Center of Brain, Behavior and Metabolism, University of Luebeck, Lübeck, Germany; 4 Joint Research Center for Computational Biomedicine (JRCC), Uniklinik RWTH Aachen University, Aachen, Germany; 5 Interdisciplinary Center for Clinical Research (IZKF), Faculty of Medicine, Research Group Neurosciences, Uniklinik RWTH Aachen University, Aachen, Germany; 6 Institute for Computational Biomedicine and Disease Modelling With Focus on Phase Transitions Between Phenotypes, Uniklinik RWTH Aachen University, Aachen, Germany

## Abstract

Voltage-gated sodium channels (VGSCs) in the peripheral nervous system shape action potentials (APs) and thereby support the detection of sensory stimuli. Most of the nine mammalian VGSC subtypes are expressed in nociceptors, but predominantly, three are linked to several human pain syndromes: while Na_v_1.7 is suggested to be a (sub-)threshold channel, Na_v_1.8 is thought to support the fast AP upstroke. Na_v_1.9, as it produces large persistent currents, is attributed a role in determining the resting membrane potential. We characterized the gating of Na_v_1.1–Na_v_1.3 and Na_v_1.5–Na_v_1.9 in manual patch clamp with a focus on the AP subthreshold depolarization phase. Na_v_1.9 exhibited the most hyperpolarized activation, while its fast inactivation resembled the depolarized inactivation of Na_v_1.8. For some VGSCs (e.g., Na_v_1.1 and Na_v_1.2), a positive correlation between ramp current and window current was detected. Using a modified Hodgkin–Huxley model that accounts for the time needed for inactivation to occur, we used the acquired data to simulate two nociceptive nerve fiber types (an Aδ- and a mechano-insensitive C-nociceptor) containing VGSC conductances according to published human RNAseq data. Our simulations suggest that Na_v_1.9 is supporting both the AP upstroke and its shoulder. A reduced threshold for AP generation was induced by enhancing Na_v_1.7 conductivity or shifting its activation to more hyperpolarized potentials, as observed in Na_v_1.7-related pain disorders. Here, we provide a comprehensive, comparative functional characterization of VGSCs relevant in nociception and describe their gating with Hodgkin–Huxley–like models, which can serve as a tool to study their specific contributions to AP shape and sodium channel–related diseases.

## Teaser

Köster et al. characterize eight sodium channel subtypes relevant to nociception and use a computer model to simulate the response of two types of nociceptive nerve fibers. Their results provide a better understanding of Na_v_1.7 and Na_v_1.9 and have implications for future mechanistic studies and disease modelling.

## Introduction

Next to their well-known role to initiate the upstroke of the action potential (AP), voltage-gated sodium channels (VGSCs) fine-tune cellular excitability by contributing to subthreshold depolarizations and fluctuations of the membrane potential. Thus, they are involved in the detection and amplification of sensory stimuli, e.g., in peripheral sensory neurons such as nociceptors (Bhattacharjee et al., 2018; Bennett et al., 2019; Goodwin and McMahon, 2021; Tian et al., 2023). Nociceptive stimuli depolarize the cell membrane of the nerve fiber ending, but if they are small, they do not initiate an AP, leading to subthreshold depolarizations. Subthreshold depolarizations directly preceding an AP are described in rats to be supported by VGSC currents sensitive to the pufferfish neurotoxin tetrodotoxin (TTX) (Enomoto et al., 2006; Vasylyev and Waxman, 2012), but the specific contribution of each channel isoform is yet to be fully understood.

To date, nine isoforms of mammalian VGSC α subunits have been described (Na_v_1.1–Na_v_1.9), which are encoded by the genes *SCN1A–SCN5A* and *SCN8A–SCN11A* (Yu and Catterall, 2003; Catterall et al., 2005; Dib-Hajj et al., 2010). They differ in their biophysical properties, such as the kinetics and voltage dependence of activation, inactivation, and deactivation (Ahern et al., 2016; Körner and Lampert, 2020; Catterall, 2023) and are classified according to their sensitivity to TTX as either TTX-resistant (TTXr; Na_v_1.5, Na_v_1.8, and Na_v_1.9) or TTX-sensitive (TTXs; all remaining isoforms) (de Lera Ruiz and Kraus, 2015).

Depending on their type and physiological function, excitable human cells express different VGSC isoforms (Catterall et al., 2005; Ahern et al., 2016). For example, Na_v_1.1–Na_v_1.3 and Na_v_1.6 are widely expressed in neurons of the central nervous system (CNS) and peripheral nervous system (PNS) (Whitaker et al., 2000; Vacher et al., 2008; Liang et al., 2021), while Na_v_1.7–Na_v_1.9 are expressed in PNS neurons, including dorsal root ganglion (DRG) neurons, sympathetic ganglion neurons, or olfactory sensory neurons (Fukuoka et al., 2008; Wang et al., 2017). The skeletal and cardiac muscles express their own specific subtypes: Na_v_1.4 and Na_v_1.5, respectively (Sheets and Hanck, 1999). Na_v_1.5 can also be detected in PNS neurons during development or injury (Renganathan et al., 2002) and at low levels in adults as revealed by recent transcriptomics studies of human sensory neurons (Tavares-Ferreira et al., 2022).

The VGSC expression patterns of different excitable cell populations can overlap and are subject to dynamic changes during development or in response to injury or sensitization. Expression of Na_v_1.3, for example, was described in embryonic, but not in healthy adult rat DRG neurons. However, expression of Na_v_1.3 and associated currents has been found to be upregulated in rodent DRG neurons after spinal cord injury (Felts et al., 1997; Hains et al., 2003; Lampert et al., 2006).

The sensation of pain via DRG neurons is associated with the activation of their afferent C- or Aδ-nerve fibers. Among them, the subclass of C-fibers known as “silent,” “sleeping,” or mechano-insensitive C-fibers (CMis) is assumed to be involved in the generation of neuropathic pain by exhibiting abnormal spontaneous activity (Namer and Handwerker, 2009; Kleggetveit et al., 2012). Cell excitability of different fiber types and therefore their function in pain signaling are subject to various factors of influence. Recent transcriptomic studies including human tissue revealed distinct VGSC expression patterns in each sensory nerve fiber type (Usoskin et al., 2015; Kupari et al., 2021; Nguyen et al., 2021; Tavares-Ferreira et al., 2022; Jung et al., 2023; Bhuiyan et al., 2024). To date, the influence of these expression patterns on nerve fiber excitability is yet to be fully understood. It is therefore beneficial to first investigate the contribution of each VGSC isoform to nerve fiber excitability in isolation.

Gain-of-function variants of Na_v_1.7 are linked to inherited pain syndromes such as erythromelalgia (Yang et al., 2004; Lampert et al., 2010; Bennett and Woods, 2014; Brouwer et al., 2014; Tang et al., 2015; Dib-Hajj et al., 2017) or paroxysmal extreme pain disorder (Fertleman et al., 2006, 2007; Jarecki et al., 2008; Stępień et al., 2020). Studies of these mutations in heterologous expression systems show a prominent shift of voltage dependence of activation to more hyperpolarized potentials and suggest that Na_v_1.7 plays an important role in the initiation of APs. Recently, however, in recordings of human sensory neurons derived from induced pluripotent stem cells (iPSC), the contribution of Na_v_1.7 to subthreshold depolarizations was observed to be limited, and the channel was considered a threshold channel rather than a subthreshold channel (Meents et al., 2019).

Functional studies in rodents and heterologous expression systems so far suggest that Na_v_1.8 is the main contributor to the upstroke of the AP and determines its duration by shaping the AP’s shoulder (Renganathan et al., 2001; Blair and Bean, 2002; Matsutomi et al., 2006; Han et al., 2015a). Na_v_1.9, on the other hand, is thought to modulate the resting membrane potential by eliciting large persistent currents (Herzog et al., 2001; Maingret et al., 2008; Tian et al., 2023). A contribution of Na_v_1.9 to the AP shoulder was nicely demonstrated in enteric neurons of Na_v_1.9 knockout mice (Osorio et al., 2014) and observed in DRG neurons of mice carrying a Na_v_1.9 missense mutation (Leipold et al., 2013), while another study failed to see an influence of Na_v_1.9 on AP shape (Priest et al., 2005). Thus, the question of the role of Na_v_1.9 in shaping AP properties is left unresolved.

Na_v_1.9 is long known to show poor heterologous expression (Vanoye et al., 2013; Goral et al., 2015; Sizova et al., 2020). Various attempts to express Na_v_1.9 in heterologous expression systems yielded limited success with very few exceptions (Tate et al., 1998; Leipold et al., 2013, 2015; Vanoye et al., 2013). Although chimeric Na_v_1.9 channels containing the C terminus from Na_v_1.4 show improved expression, they exhibit altered voltage dependence of channel activation and inactivation (Goral et al., 2015; Bothe and Lampert, 2021) and are therefore unsuitable for studying Na_v_1.9 gating and its contribution to AP genesis.

Once the gating parameters of each channel subtype have been analyzed, their contribution to cellular excitability can be reconstructed and investigated using mathematical and in silico models. In 1952, Hodgkin and Huxley provided a mathematical description of VGSC gating and APs, which still today is often used to model electrophysiological data and cellular excitability (Hodgkin and Huxley, 1952; Stiles and Gray, 2021; Wang et al., 2022). However, classic Hodgkin–Huxley models implement VGSC inactivation right at the beginning of a voltage step stimulus and therefore do not account for the duration of protein-level conformational changes that are assumed to occur within the channel for the so-called inactivation particle to bind to its receptor and thereby stop the associated sodium influx.

C-fiber excitability in particular has been modelled and used successfully to describe the biophysical properties of this fiber type (Petersson et al., 2014; Tigerholm et al., 2014, 2015; Maxion et al., 2023). In most cases, only select VGSC isoforms are included in models of excitable cells, often because of a lack of a standardized comprehensive data set, which is needed as a basis for model generation. Larger studies comparing VGSC gating in vitro in a comparable experimental setting have focused on suprathreshold gating of a subset of VGSCs expressed in the CNS and related mutations leading, e.g., to epilepsy (Thompson et al., 2023), or on sensory neuron VGSCs with a focus on their temperature dependence (Kriegeskorte et al., 2023).

As conclusive and complete data on the sub- and suprathreshold activity of all VGSC isoforms are not yet available, we here conducted a standardized and comparative examination of VGSC isoforms involved in nociception (Na_v_1.1–1.3 and Na_v_1.5–1.8) with consistent experimental conditions using patch-clamp experiments in heterologous expression systems (HEK293(T) and ND7/23 cells).

With these data, we developed a computational model which is based on the Hodgkin–Huxley framework and parameterized using our patch-clamp data. We added Na_v_1.9 to this model using previously published data obtained under similar experimental conditions using transfected ND7/23 cells (Leipold et al., 2013, 2015). We implemented the VGSC isoform proportions as reported for human CMis and Aδ-fibers (Tavares-Ferreira et al., 2022) to investigate the contributions of individual VGSC isoforms to excitability and AP generation in two sensory fiber types.

## Materials and methods

### Cell culture and cell preparation

#### Cell lines

Plasmids of VGSC isoforms used in this study were either stably transfected in the human embryonic kidney cell line HEK293 (rat Na_v_1.3 = rNa_v_1.3, human Na_v_1.5 = hNa_v_1.5, and mouse Na_v_1.6 = mNa_v_1.6, hNa_v_1.7) or transiently in the human embryonic kidney cell line HEK293T (hNa_v_1.1, hNa_v_1.2) or mouse/rat hybridoma nerve cell line ND7/23 (hNa_v_1.8).

All used cell lines were cultivated adherently under standard cell culture conditions (i.e., 37°C and 5% CO_2_) in their respective cell culture media and supplements (Table S1). Passaging for all cell lines was performed twice per week at ∼80% confluency using Accutase (Sigma-Aldrich) with a 1:5–1:10 passaging rate onto cultureware coated with Geltrex (LDEV-Free, hESC-Qualified; Gibco, Thermo Fisher Scientific). Only cells with passage numbers below 30 were used for electrophysiological experiments. All cell lines were regularly tested negative for mycoplasma contamination.

#### Plasmids

Plasmid constructs used for transient transfection were amplified using XL10-gold ultracompetent cells (Agilent Technologies, Inc.) and verified by restriction pattern analysis and commercial DNA sequencing (Eurofins Genomics GmbH). The plasmids for hNa_v_1.1 (pCMV6-AC-hNa_v_1.1 WT RRSSV 3′ UTR, Myc-DDK) and hNa_v_1.2 (pCMV6-XL5-hNa_v_1.2 WT RRSSV 3′ UTR) were kindly gifted by Frank Bosmans, Ghent University, Belgium. hNa_v_1.1 contained the common natural variation A1067T (allele frequency for T: 0.728). The hNa_v_1.8 plasmid (pIRES puro3-hNa_v_1.8 WT) contained the V1073A variant (allele frequency for A: 0.58).

#### Transient transfection

HEK293T and ND7/23 cells were seeded in 35-mm petri dishes 20–24 h prior to transient transfection to reach a 70–90% confluency. Transfection was then performed using 3 μl jetPEI (Polyplus-transfection), 0.25 µg reporter plasmid pMax-GFP (Lonza), and 1.25 µg plasmid encoding the VGSC to be examined. Cells were incubated for another 18–24 h before being split onto fresh 35-mm petri dishes. About 2 h later, after the seeded cells attached to the cultureware, cells with a bright green fluorescence were used for patch-clamp experiments.

### Electrophysiology

#### General patch-clamp framework

Electrophysiological experiments were performed in whole-cell voltage-clamp mode using an EPC10-USB amplifier (HEKA Elektronik GmbH). Cells were covered in extracellular solution (ECS) containing 140 mM NaCl, 3 mM KCl, 1 mM MgCl_2_, 1 mM CaCl_2_, 10 mM HEPES, and 20 mM glucose. ECS was adjusted to pH 7.4 using NaOH, while osmolarity was between 305 and 312 mosm/liter. Since ND7/23 cells display endogenous VGSC-mediated TTXs currents (Rogers et al., 2016; Lee et al., 2019), 500 nM TTX (Tocris) was added to the ECS for measurements of the TTXr channel hNa_v_1.8.

Glass pipettes from borosilicate glass tubes (Biomedical Instruments) were manufactured and fire-polished using a DMZ pipette puller (Zeitz Instruments GmbH). Pipette tip resistance was between 0.8 and 2 MΩ for all measurements. The pipette was filled with intracellular solution (ICS) containing 10 mM NaCl, 140 mM cesium fluoride (CsF), 1 mM EGTA, 10 mM HEPES, and 18 mM sucrose. ICS was adjusted to pH 7.31 with CsOH, while osmolarity was 313 mosm/liter.

Experiments were conducted at room temperature (22 ± 1°C). Cells were kept in ECS for a maximum of 90 min before being discarded. Cell membrane capacitance was between 5 and 33 pF. For all measurements, seal resistance was above 0.8 GΩ. Only cells with an initial series resistance (R_s_) below 5 MΩ and a peak current amplitude above 500 pA during the voltage dependence of the activation protocol were accepted. At all times, voltage error (R_s_ * I) was below 5 mV.

Data were gathered using Patchmaster Next version 1.2 (HEKA Elektronik GmbH). Analog signals were digitized at a sampling rate of 100 kHz (except for AP clamping measurements, which were sampled at 20 kHz) with a 10-kHz Bessel low-pass filter. Leak currents were measured and subtracted online (for activation, steady-state fast inactivation [SSFI], and ramp current measurements) or offline (for AP clamping due to technical limitations) with a *P/n* procedure (*n* = 4) preceding the respective test pulse. Capacitive currents were cancelled, and R_s_ was compensated for by 77–83% for all cells. All measurements were corrected online for a liquid junction potential of 8.2 mV.

#### Patch-clamp protocols

For each cell, stimulation protocols were only recorded once (no technical replicates). Protocols are indicated in the respective figures. For all protocols, the holding potential in between test pulses (V_hold_) was set to −120 mV.

After establishing whole-cell configuration, a current stabilization protocol was conducted for 5 min to ensure complete recovery, in which cells were held at holding potential and repeatedly stimulated with test pulses to 0 mV every 10 s.

To investigate the voltage dependence of activation, a series of 40-ms pulses from holding potential to a test pulse potential was elicited with the test pulse potential increasing stepwise from −90 to +40 mV in 10-mV increments (−60 to +70 mV for most Na_v_1.8 measurements). The interpulse interval was 5 s. For assessment of the voltage dependence of activation (i.e., the fraction of channels activated to an open state depending on the membrane potential), we calculated the voltage-dependent sodium conductance (*G*_*Na*_) for each test pulse using the following equation:

$$G_{Na}=\frac{I_{Na}}{V_{m}-E_{rev}}$$ (1)

with *I*_*Na*_ being the peak inward current at the respective test pulse voltage *V*_*m*_, and *E*_*rev*_ being the sodium reversal potential individually determined for each cell. To exclude effects of varying cell size and current density, *G*_*Na*_ values were normalized to the maximum conductance *G*_*Na*,*max*_ of that cell before being plotted against test pulse voltages.

During the voltage dependence of the SSFI protocol, after being held at holding potential, cells were initially stimulated with a 500-ms prepulse before being exposed to a 40-ms test pulse at 0 mV. From sweep to sweep, the prepulse voltage increased stepwise from −140 to −30 mV in 10-mV increments (−110 to 0 mV for most Na_v_1.8 measurements). The interpulse interval was 10 s. For investigation of the voltage dependence of SSFI (i.e., the fraction of non-inactivated channels available for activation depending on the preceding membrane potential), the peak inward current for each test pulse (*I*_*Na*_) was normalized to the maximum inward current during inactivation measurement (*I*_*Na*,*max*_). Normalized current values were plotted against the respective prepulse potential.

Voltage dependence of activation conductance-voltage curves and voltage dependence of SSFI current-voltage curves were fitted with nonlinear regression using the Boltzmann equation:

$$\frac{G_{Na}}{G_{Na,\max}}\;\vee \;\frac{I_{Na}}{I_{Na,\max}}=bottom+\frac{top-bottom}{1+e^{\frac{V_{50}-V_{m}}{k}}}$$ (2)

with *V*_*50*_ being the membrane potential at half-maximal channel (in)activation in mV, *V*_*m*_ being the membrane voltage in mV, and *k* being the slope factor in 1/mV. The top value of the sigmoid curve was constrained to 1 for both activation and inactivation measurements. The bottom value was constrained to 0 for activation measurements but was not constrained for inactivation measurements to examine the fraction of channels remaining open after SSFI. The goodness of fit was *R*^*2*^ > 0.9 for all Boltzmann fits.

We equated the window current for each cell as the area under the curve (AUC) of the superimposed activation and SSFI Boltzmann fit curves. Because SSFI Boltzmann fits do not reach 0 values when approaching infinity voltages, the AUC was calculated with an upper limit set 20 mV above the intersection of activation and SSFI Boltzmann fits for each cell.

For ramp current measurements, cells were stimulated with depolarizing voltage ramps from holding potential to +20 mV at different ramp rates, namely 0.1, 0.2, 0.4, 0.6, 1.2, 2, 4, and 6 mV/ms (for ramp durations see Table S2). For each ramp rate, the measured current response was averaged from three consecutive sweeps. The maximum inward current and the voltage at which it occurs were measured, as well as the AUC of the current response. Because especially for Na_v_1.8, the ramp current bell curve did not return to 0 pA at the end of the ramp, the AUC was calculated only up to the maximum inward current. Maximum inward current and AUC were normalized to the respective maximum inward current from the activation protocol for each cell (*I*_*Na*,*max*_).

To measure the VGSC current response to APs, three different pre-recorded APs (AP1–AP3) measured in iPSC-derived nociceptors were used as a voltage command (Blair and Bean, 2002). The AP commands differ in their characteristics (Table 1) with, e.g., subthreshold voltage slope being between 0.2 and 2 mV/ms. These voltage stimuli included 50 ms of the respective resting membrane potential preceding each recorded AP (Table 1) to prevent transient currents caused by step depolarization from holding potential to initial AP voltage from interfering with the examined AP current responses. The respective AP current response was averaged out of three consecutive sweeps. For each stimulation, we calculated the maximum inward current and AUC of both the total AP and of the subthreshold phase of the AP. For the latter, the slope of the elicited current was calculated as well. Current responses for AP clamping were normalized to the maximum inward current during activation measurement (*I*_*Na*,*max*_).

**Table 1.** Properties of the pre-recorded APs used as voltage commands

	AP1	AP2	AP3
Average resting membrane potential (mV)	−76.5	−71.9	−67.4
AP threshold voltage (mV)	−60.1	−61.2	−41
Maximum AP voltage (mV)	10	11.2	59.3
Voltage amplitude of AP (mV)	86.5	83.1	126.7
Minimum voltage of after hyperpolarization (mV)	−71.6	−67.7	−59.7
AP half width (ms)	3.6	2.4	3.8
Time to AP peak (ms)	99.6	14.6	18
Subthreshold slope (mV/ms)	0.21	1.39	1.84
Upstroke slope (mV/ms)	3.31	10.64	27.1

Data were gathered from current-clamp measurements of iPSC-derived nociceptors using the Igor Pro software.

### Sample sizes and data exclusion

We performed an a priori analysis to estimate the number of cells needed for each experiment. In a small pre-study with Na_v_1.3 and Na_v_1.7, the voltage at which the maximum inward ramp current appeared was compared, assuming normal distribution. Using the software G*Power version 3.1.9.7 (Heinrich-Heine-Universität), we calculated the sample size for each VGSC to be *n* = 27 (hedge’s g* = 1.23 as a measure of the effect size *d*, two-tailed *α* = 0.05 and power [1−*β*] = 0.95).

We designed the study to have groups of equal size. Group size variation might occur due to cells being excluded from analysis a posteriori, e.g., because of measurement artifacts impeding data analysis. Since the ramp current responses often showed a considerable drift, which made it difficult to calculate the AUC, the following exclusion criteria were defined for ramp current AUC measurements: cells with a drift larger than +100 or −500 pA as well as cells with a drift >10% of the maximum inward current during activation measurement were excluded from analysis. For AP clamping measurements, we identified outliers using the ROUT method with *Q* = 0.1% (Motulsky and Brown, 2006). Outliers were excluded from further analysis. Beyond that, we performed no outlier analysis or elimination.

### Data analysis

Data analysis for electrophysiological experiments was blinded except for Na_v_1.5. Data gathered by electrophysiological experiments were analyzed using FitMaster version 2 × 90.5 (HEKA Electronik GmbH) and IgorPro version 6.37 (WaveMetrics, Inc.). IgorPro analysis procedures are available upon reasonable request. GraphPad Prism version 9.5.1 (GraphPad Software) was used for Boltzmann fits and statistical testing. Graphing of data was performed with IgorPro, GraphPad Prism, and CorelDRAW 2017 version 19.0.0.328 (Alludo).

### Statistics

Data are presented as mean ± SD unless stated otherwise. Error bars denote the SD, while box plot whiskers show the 10th and 90th percentiles. The threshold for statistical significance was set to P < 0.05. Given P values are shown with four decimal places and summarized as follows: ns = not significant, * = P < 0.05, ** = P < 0.01, and *** = P < 0.001.

Data were tested for normal distribution using the D’Agostino & Pearson omnibus K2 test. Normal distribution for an experiment was only assumed if each group passed the test individually. For statistical testing, groups were compared by ordinary one-way ANOVA with Tukey–Kramer’s multiple comparisons test and a single-pooled variance for parametric testing or Kruskal–Wallis’s test with Dunn’s multiple comparison test for nonparametric testing. Values derived from ramp current measurements (*I*_*max*_, *U*_*location of Imax*_, and AUC) were compared by ordinary two-way ANOVA with Tukey–Kramer’s multiple comparisons test, and individual variances were computed for each comparison to compare between both VGSC isoforms and an additional factor of influence, i.e., the ramp rate. For correlations, we computed the nonparametric Spearman correlation with a two-tailed P value (data are given as Spearman’s *r* with 95% CI) and graphed it with simple linear regression. Data were neither matched nor paired.

The exact group *n* values are not shown in the figures for readability purposes but are indicated in the respective tables and supplementary tables.

### Computational modelling

To simulate the AP of a CMi and an Aδ-fiber, we use a modified version of the Hodgkin–Huxley model. Compared with the original Hodgkin–Huxley model (Hodgkin and Huxley, 1952), we introduced a delay for current decay (fast inactivation kinetics). This modification prevents premature inactivation of sodium channels and improves the fit of the model to sodium currents of Na_v_1.1–Na_v_1.3 and Na_v_1.5–Na_v_1.8 for activation voltages above 10 mV. The gating variables and the delay of inactivation were fitted using voltage-clamp data for Na_v_1.1–Na_v_1.3 and Na_v_1.5–Na_v_1.8 acquired at room temperature (22 ± 1°C) as well as previously published voltage-clamp data for Na_v_1.9 (Leipold et al., 2013, 2015). To fit Na_v_1.9 kinetics, the original Hodgkin–Huxley equations were used because they accurately captured the data. The fits were performed with fmincon from the MATLAB software R2021b (The MathWorks, Inc.) using a multistart approach with Latin hypercube sampling and a weighted least squares cost functional. To simulate APs in a CMi and an Aδ-fiber, the ratios between the maximal conductances for the considered VGSC isoforms were chosen to match recently published putative gene expressions quantified in human DRGs by spatial transcriptomics (Tavares-Ferreira et al., 2022). The simulations are based on the quantifications of gating variables at room temperature. The resting potential is −71 mV, and the model is at rest before different stimulation currents are applied. Details, model equations, and parameters are provided in the supplementary information.

### Online supplemental material

**Figure S1.**
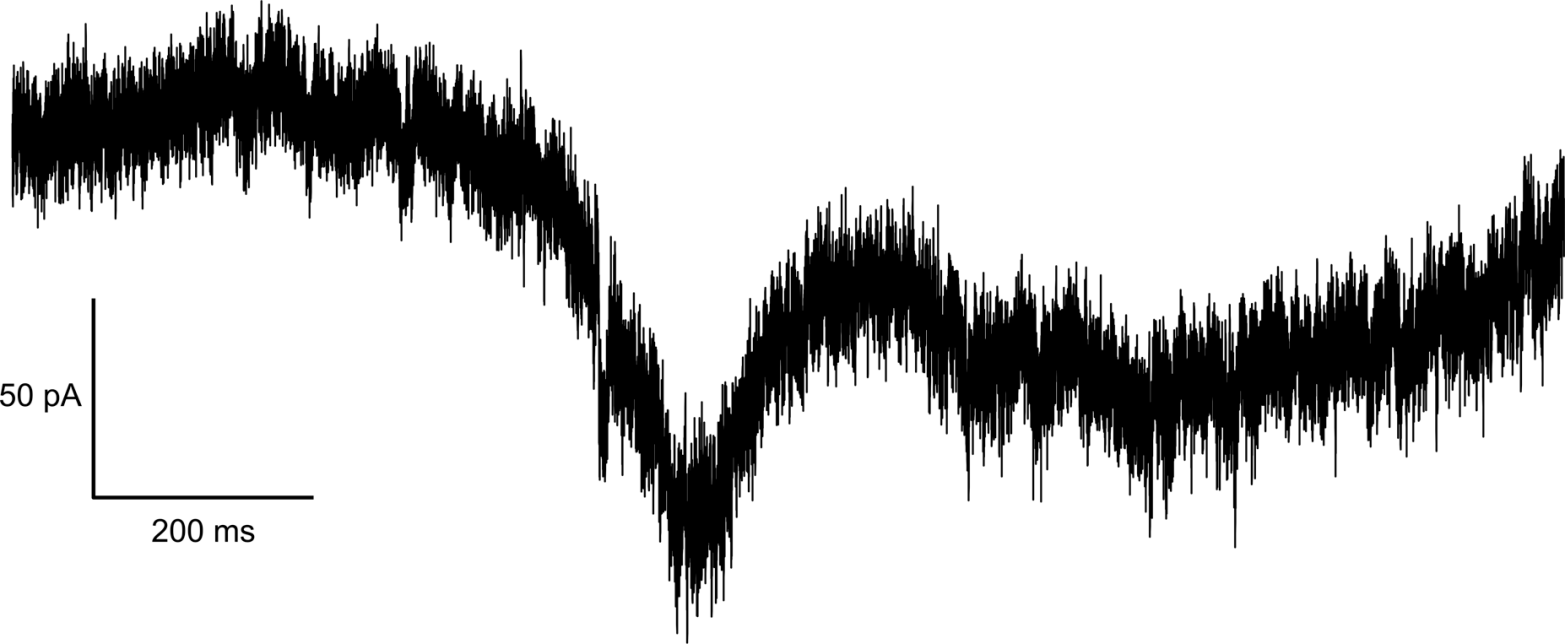
**Example trace of a Na**_**v**_ **1.3 ramp current elicited by a 0.1 mV/ms voltage ramp exhibiting two peaks.**

**Figure S2.**
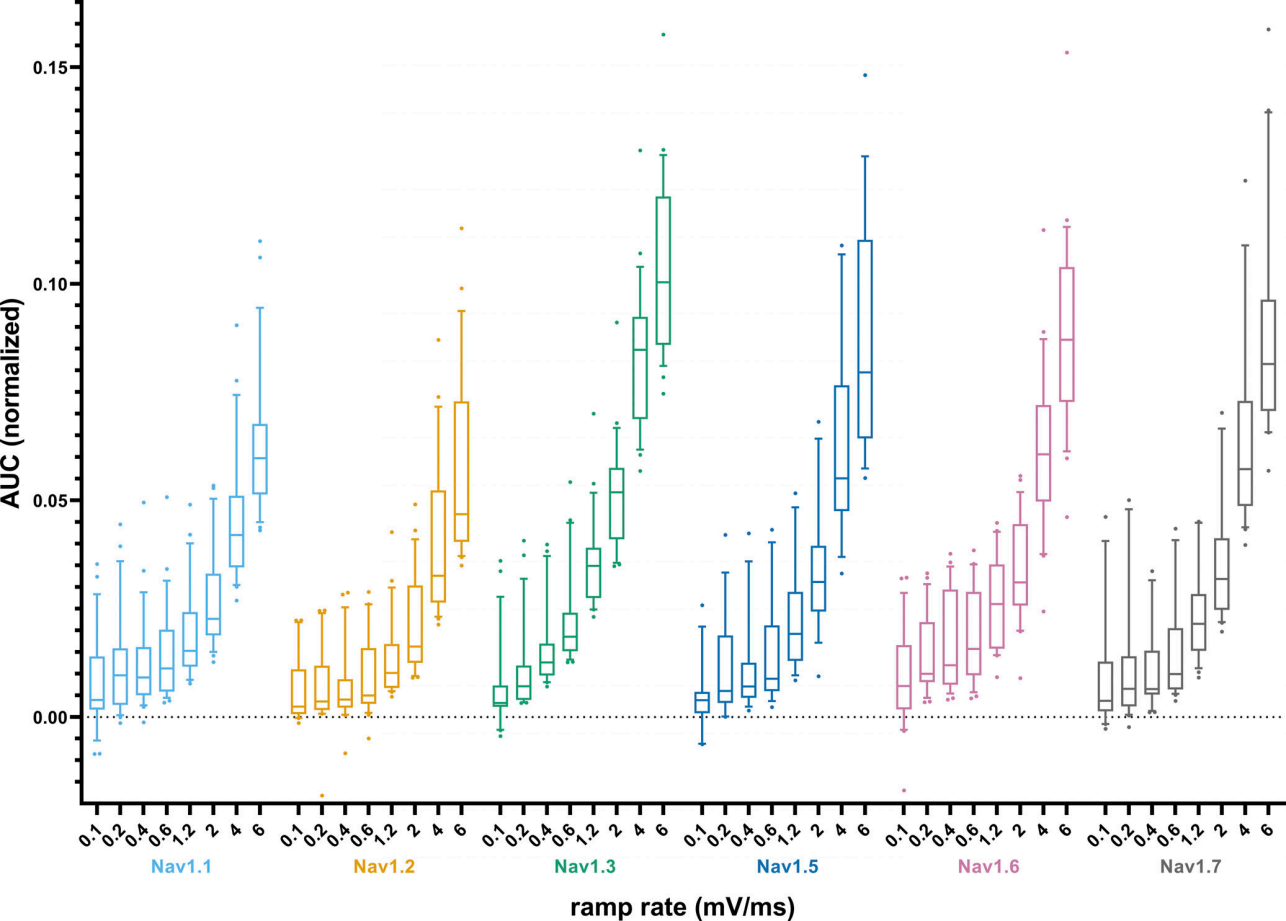
**AUC values obtained from full ramp current measurements, normalized to the maximum inward current from activation measurements for each cell.** Na_v_1.8 was left out since its bell-shaped ramp current response curves are cut at +20 mV. Data are shown as box plots with whiskers indicating the 10th and 90th percentile.

**Figure S3.**
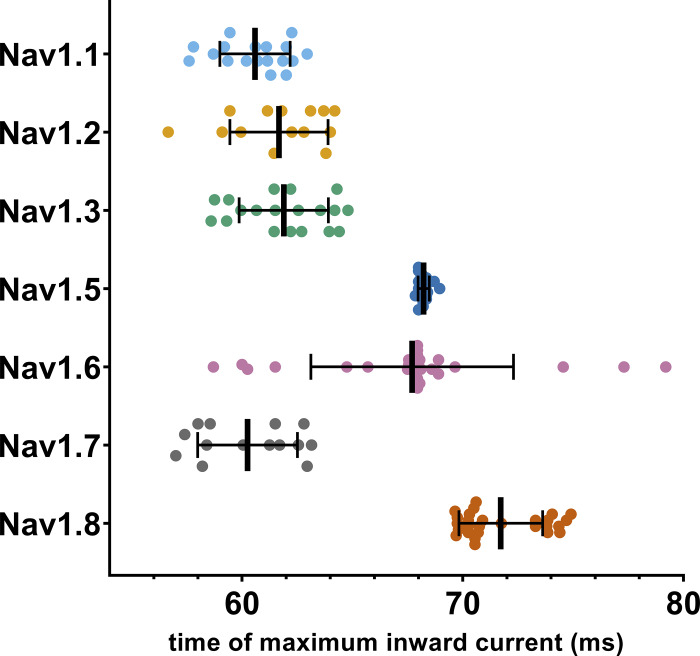
Time point at which the maximum inward current during AP3 after exclusion of measurements impeded by transient current artifacts.

**Figure S4.**
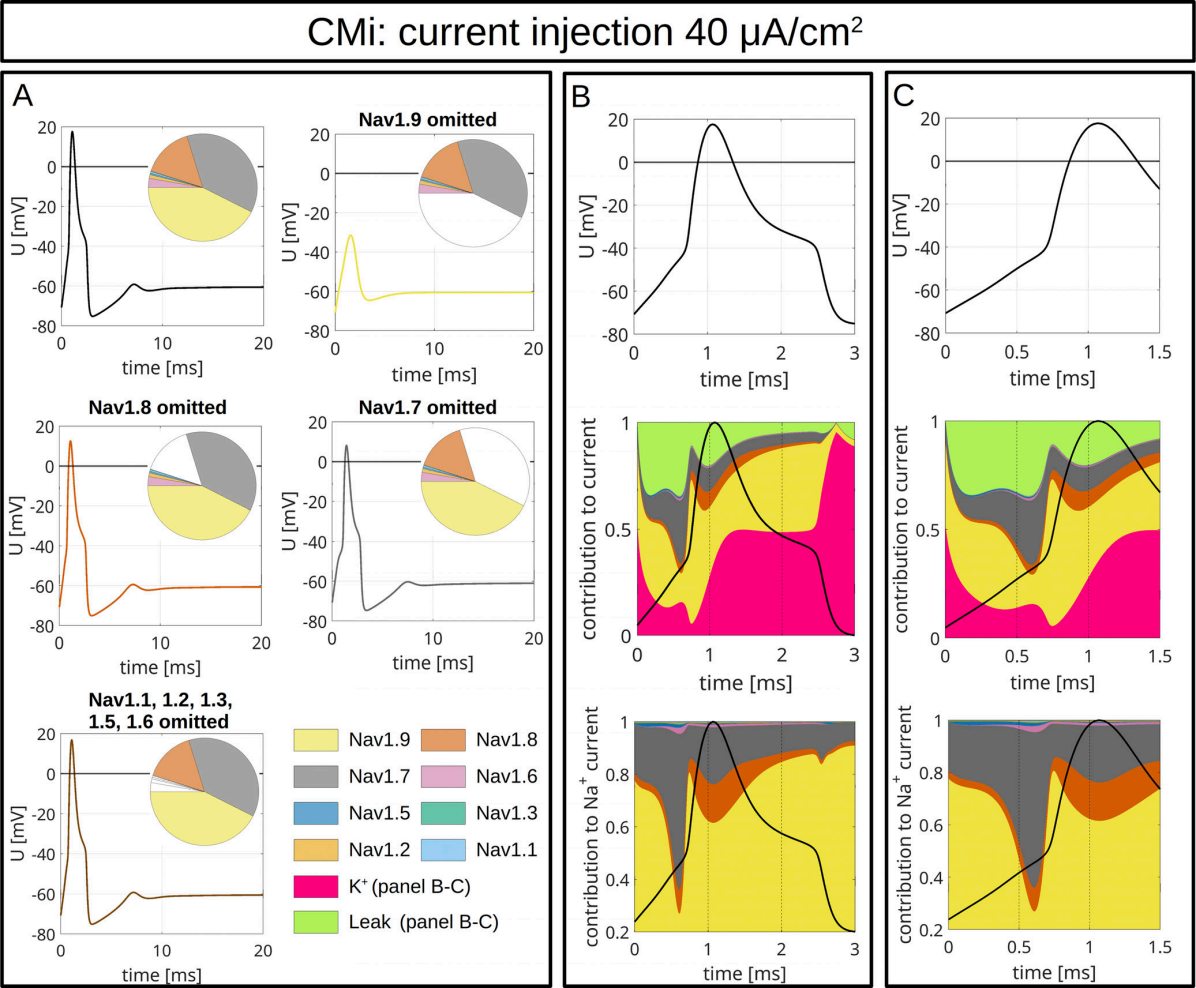
**Computer simulations of APs in a CMi.** The pie charts indicate the abundance of different VGSC isoforms. APs are triggered by an injection of 40 μA/cm^2^, which persists until the end of the simulation. **(A)** The upper left panel shows the APs for the relative abundance of VGSC isoforms quantified in spatial gene expression. The initial condition of the simulation is the resting state of the CMi. The other panels show changes of the AP resulting from removal of Na_v_1.9 (upper right), Na_v_1.8 (middle left), Na_v_1.7 (middle right), and Na_v_1.1–1.3, 1.5, and 1.6 (bottom left). The VGSC isoforms are coded by the colors specified in the bottom right panel. **(B and C)** VGSC isoform contributions to the simulated AP depicted in the upper left panel of A. The upper panels of B and C show the membrane potential during the initial 3 ms (B) and 1.5 ms (C) of the simulated AP. The middle panels show the relative contribution of the different sodium currents, the potassium current, and the leak current over the course of the AP, plotted as stacked individual currents normalized to total current at each time point. The bottom panels show VGSC isoform contributions to the total sodium current, plotted as stacked individual VGSC isoform currents normalized to the total sodium current at each time point. The black line visualizes the shape of the AP. The color coding is identical to that in A.

**Figure S5.**
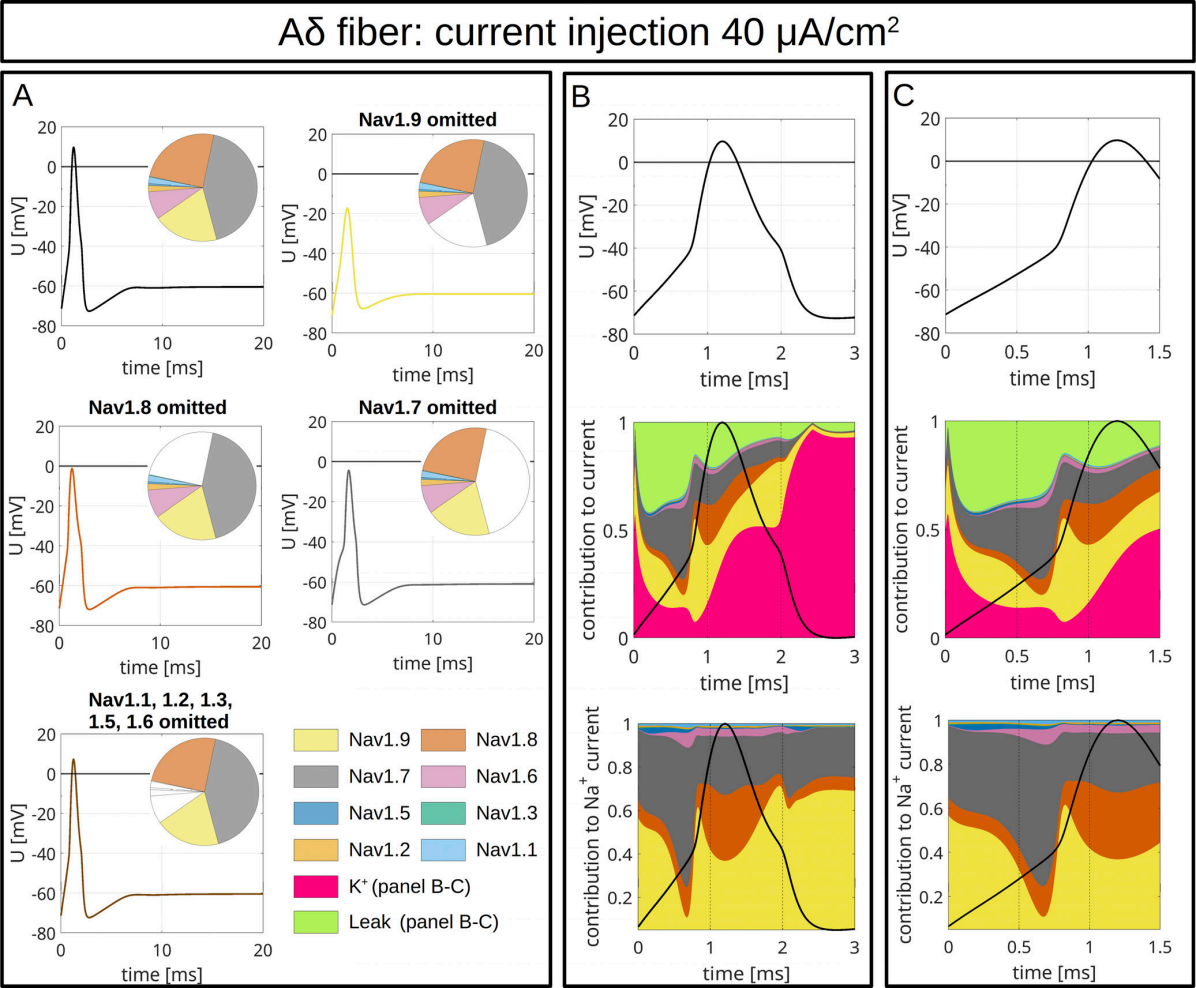
**Computer simulations of AP in an Aδ-fiber.** The pie charts indicate the abundance of different VGSC isoforms. APs are triggered by an injection of 40 μA/cm^2^, which persists until the end of the simulation. **(A)** The upper left panel shows the APs for the relative abundance of VGSC isoforms quantified in spatial gene expression. The initial condition of the simulation is the resting state of the Aδ-fiber. The other panels show changes of the AP resulting from removal of Na_v_1.9 (upper right), Na_v_1.8 (middle left), Na_v_1.7 (middle right), and Na_v_1.1–1.3, 1.5, and 1.6 (bottom left). The VGSC isoforms are coded by the colors specified in the bottom right panel. **(B and C)** VGSC isoform contributions to the simulated AP depicted in the upper left panel of A. The upper panels of B and C show the membrane potential during the initial 3 ms (B) and 1.5 ms (C) of the simulated AP. The middle panels show the relative contribution of the different sodium currents, the potassium current, and the leak current over the course of the AP, plotted as stacked individual currents normalized to total current at each time point. The bottom panels show VGSC isoform contributions to the total sodium current, plotted as stacked individual VGSC isoform currents normalized to the total sodium current at each time point. The black line visualizes the shape of the AP. The color coding is identical to that in A.

**Figure S6.**
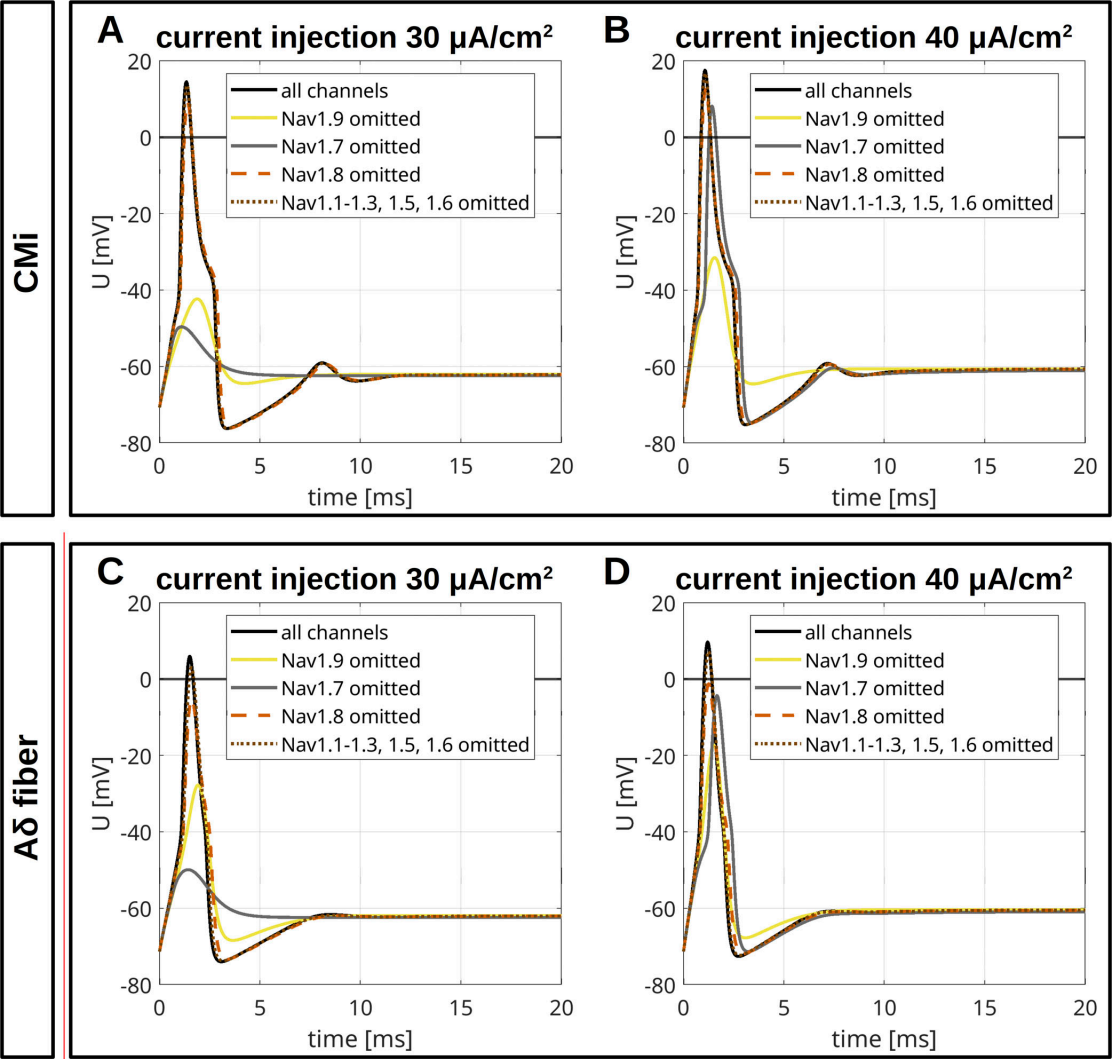
**Overlay of simulated APs in a CMi and in an Aδ-fiber. (A and B)** AP for the CMi with the relative abundance of VGSC isoforms quantified in spatial gene expression (black, isoform abundances as in Fig. 9 A, upper left panel) and changes of the AP resulting from removal of Na_v_1.9 (yellow), Na_v_1.8 (orange), Na_v_1.7 (gray), and Na_v_1.1–1.3, 1.5, and 1.6 (brown). The current injections of 30 μA/cm^2^ (A) and 40 μA/cm^2^ (B) persist until the end of the simulation. **(C and D)** AP for the Aδ-fiber with the relative abundance of VGSC isoforms quantified in spatial gene expression (black, isoform abundances as in Fig. 10 A, upper left panel) and changes of the AP resulting from removal of Na_v_1.9 (yellow), Na_v_1.8 (orange), Na_v_1.7 (gray), and Na_v_1.1–1.3, 1.5, and 1.6 (brown). The current injections of 30 μA/cm^2^ (C) and 40 μA/cm^2^ (D) persist until the end of the simulation.

**Figure S7.**
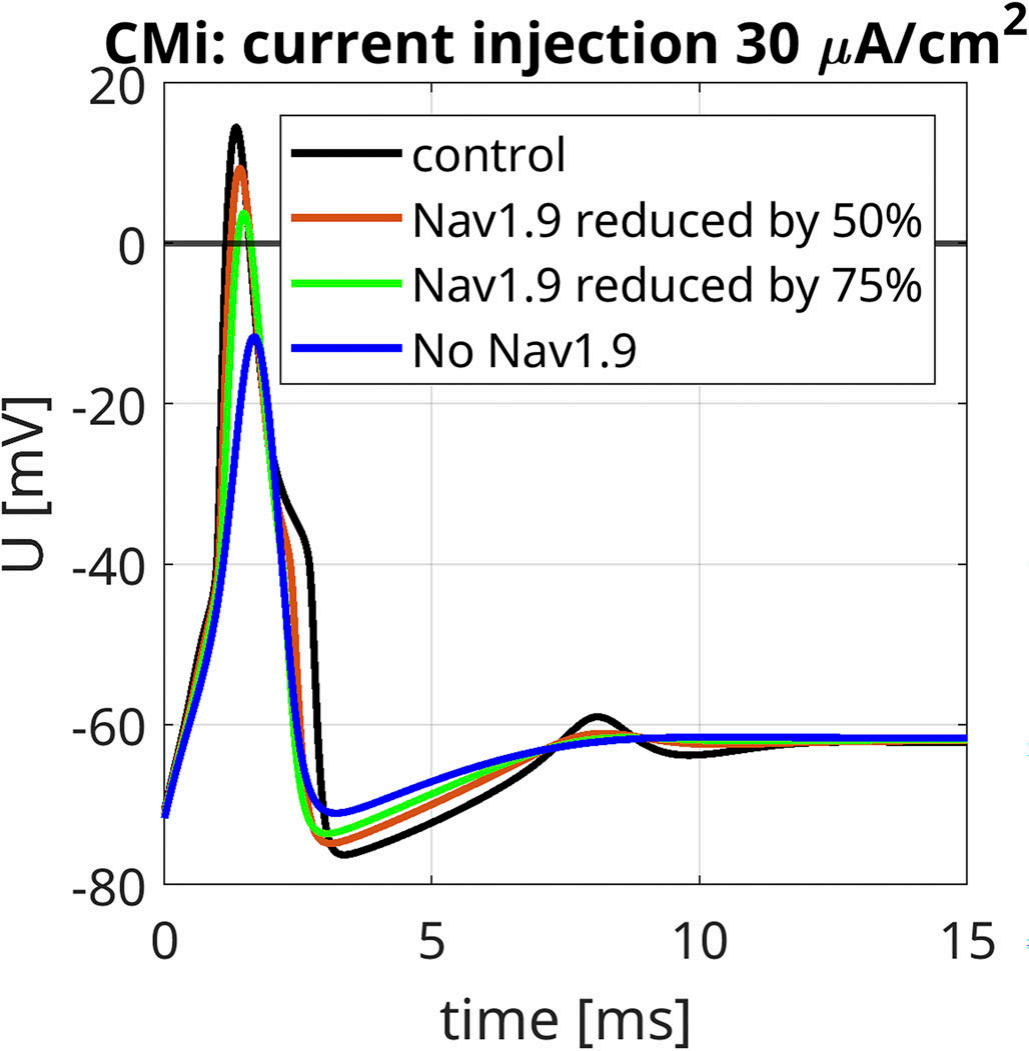
**Simulated contribution of Na**_**v**_ **1.9 to APs in a CMi.** The black line (control) shows the AP in a CMi with the relative abundance of VGSC isoforms quantified in spatial gene expression (isoform abundances as in Fig. 9 A, upper left panel). The maximal conductance of Na_v_1.9 is stepwise reduced to 50% (red line), 25% (green line), and 0% (blue line) compared with the control scenario. The maximal conductances of the other isoforms are increased proportionally to their expression, such that the total maximal *G*_*Na*_ remains unchanged. This is different to Fig. 9 A, where the total maximal *G*_*Na*_ decreased upon removal of a VGSC isoform. The current injections of 30 μA/cm^2^ persist until the end of the simulation.

**Figure S8.**
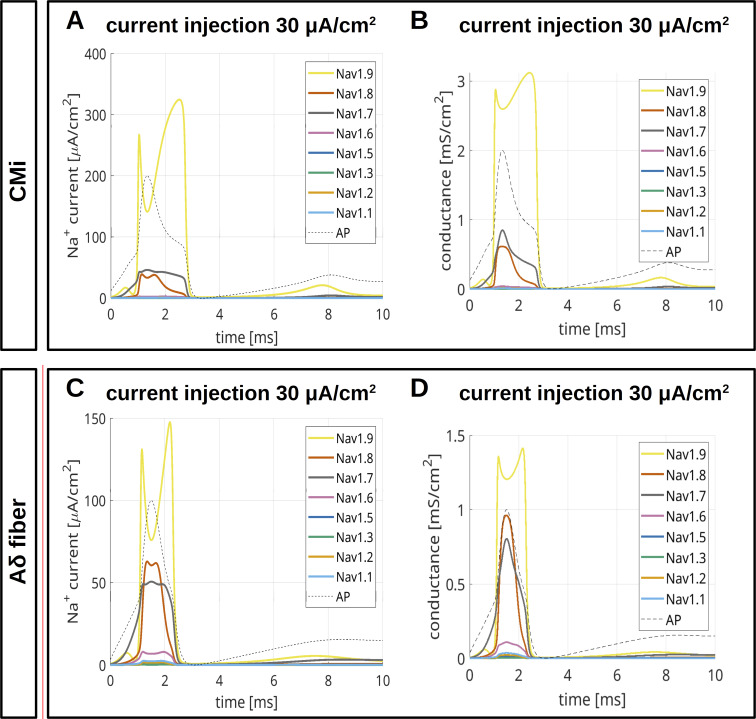
**Simulated sodium currents and conductances in a CMi and Aδ-fiber. (A)** Absolute sodium currents over the course of the simulated AP of the CMi depicted in Fig. 9 A, upper left panel. **(B)** Conductances of the different VGSC isoforms over the course of the simulated AP of the CMi depicted in Fig. 9 A, upper left panel. **(C)** Absolute sodium currents over the course of the simulated AP of the Aδ-fiber depicted in Fig. 10 A, upper left panel. **(D)** Conductances of the different VGSC isoforms over the course of the simulated AP of the Aδ-fiber depicted in Fig. 10 A, upper left panel. The current injections of 30 μA/cm^2^ persist until the end of the simulation. The dotted black line visualizes the time course of the membrane potential during the AP.

Fig. S1 shows an example trace of a Na_v_1.3 ramp current elicited by a 0.1 mV/ms voltage ramp exhibiting two peaks. Fig. S2 shows AUC values obtained from full ramp current measurements, normalized to the maximum inward current from activation measurements for each cell. Fig. S3 shows the time points at which the maximum inward current during AP3 after exclusion of measurements impeded by transient current artifacts. Fig. S4 shows computer simulations of APs in a CMi. Fig. S5 shows computer simulations of AP in an Aδ-fiber. Fig. S6 shows an overlay of simulated APs in a CMi and in an Aδ-fiber. Fig. S7 shows the simulated contribution of Na_v_1.9 to APs in a CMi. Fig. S8 shows simulated sodium currents and conductances in a CMi and Aδ-fiber. Table S1 shows an overview of all used cell lines and their culture media and supplements. Table S2 shows the duration of command voltage ramps. Table S3 shows multiple comparisons of V50 values of voltage dependency of activation and fast inactivation measurements. Table S4 shows multiple comparisons of slope values (k) of voltage dependency of activation and fast inactivation measurements. Table S5 shows multiple comparisons of window current AUC and intersection voltage of activation and SSFI Boltzmann fit curve. Table S6 shows multiple comparisons of fraction of channels remaining open after SSFI. Table S7 shows descriptive statistics of parameters gathered from ramp current measurements. Table S8 shows multiple comparisons of normalized maximum inward current values from ramp current measurements. Table S9 shows multiple comparisons of the voltage dependence of maximum inward current from ramp current measurements. Table S10 shows multiple comparisons of normalized AUC before maximum inward current values from ramp current measurements. Table S11 shows multiple comparisons of normalized AUC before maximum inward current values from ramp current measurements. Table S12 shows the correlation of window current values and AUC values from ramp current measurements. Table S13 shows descriptive statistics of parameters gathered from AP clamping measurements. Table S14 shows multiple comparisons of parameters calculated from AP clamping measurements. Appendix S1 provides data on the in silico models.

## Results

### Na_v_1.5 and Na_v_1.8 activate and fast inactivate at more hyper- and depolarized potentials than other subtypes, respectively

**Figure 1.**
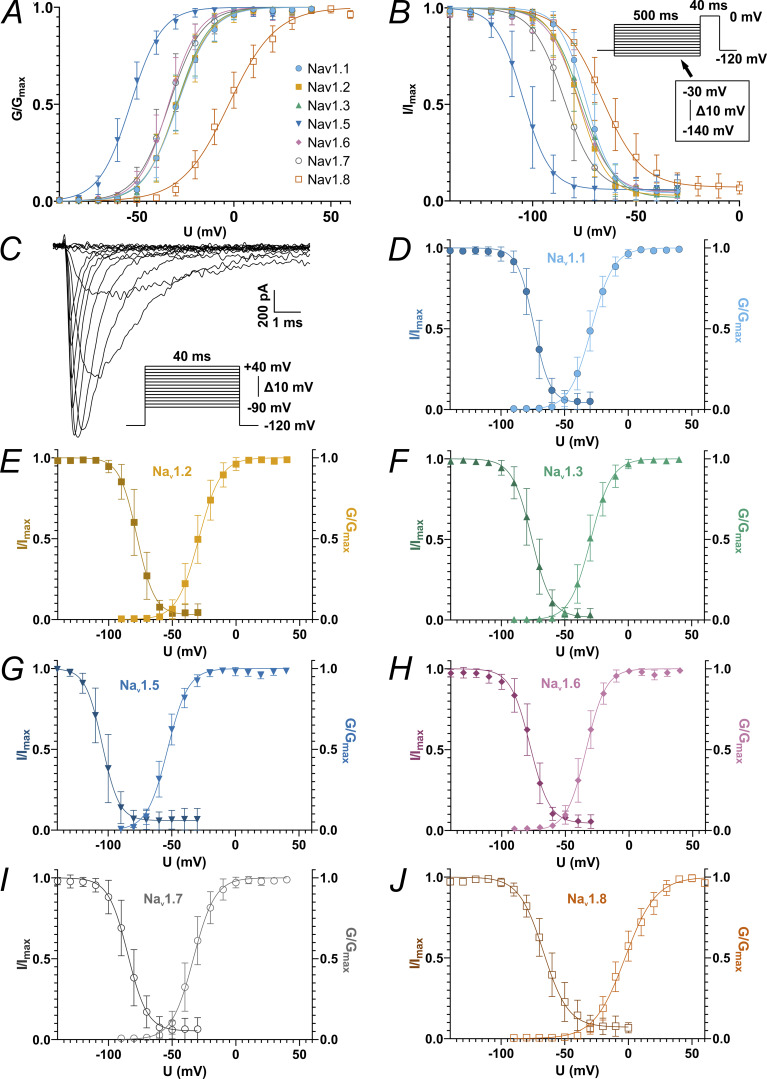
**Conductance-voltage or current-voltage relationship of activation and SSFI protocols. (A)** Overview of all conductance-voltage curves derived from activation measurements. **(B)** Overview of all current-voltage curves derived from SSFI measurements (voltage protocol of SSFI depicted in inlet. Note that for Na_v_1.8 measurements, most cells were stimulated with a −110 to 0 mV voltage range). **(C)** Example current traces of Na_v_1.7 elicited by the activation protocol (voltage protocol depicted in inlet. Note that for Na_v_1.8 measurements, most cells were stimulated with a −60 to +70 mV voltage range). **(D–J)** Overlay of activation and inactivation curves for Na_v_1.1 (D), Na_v_1.2 (E), Na_v_1.3 (F), Na_v_1.5 (G), Na_v_1.6 (H), Na_v_1.7 (I), and Na_v_1.8 (J). Data are shown as mean ± SD and superimposed Boltzmann fit curve.

**Figure 2.**
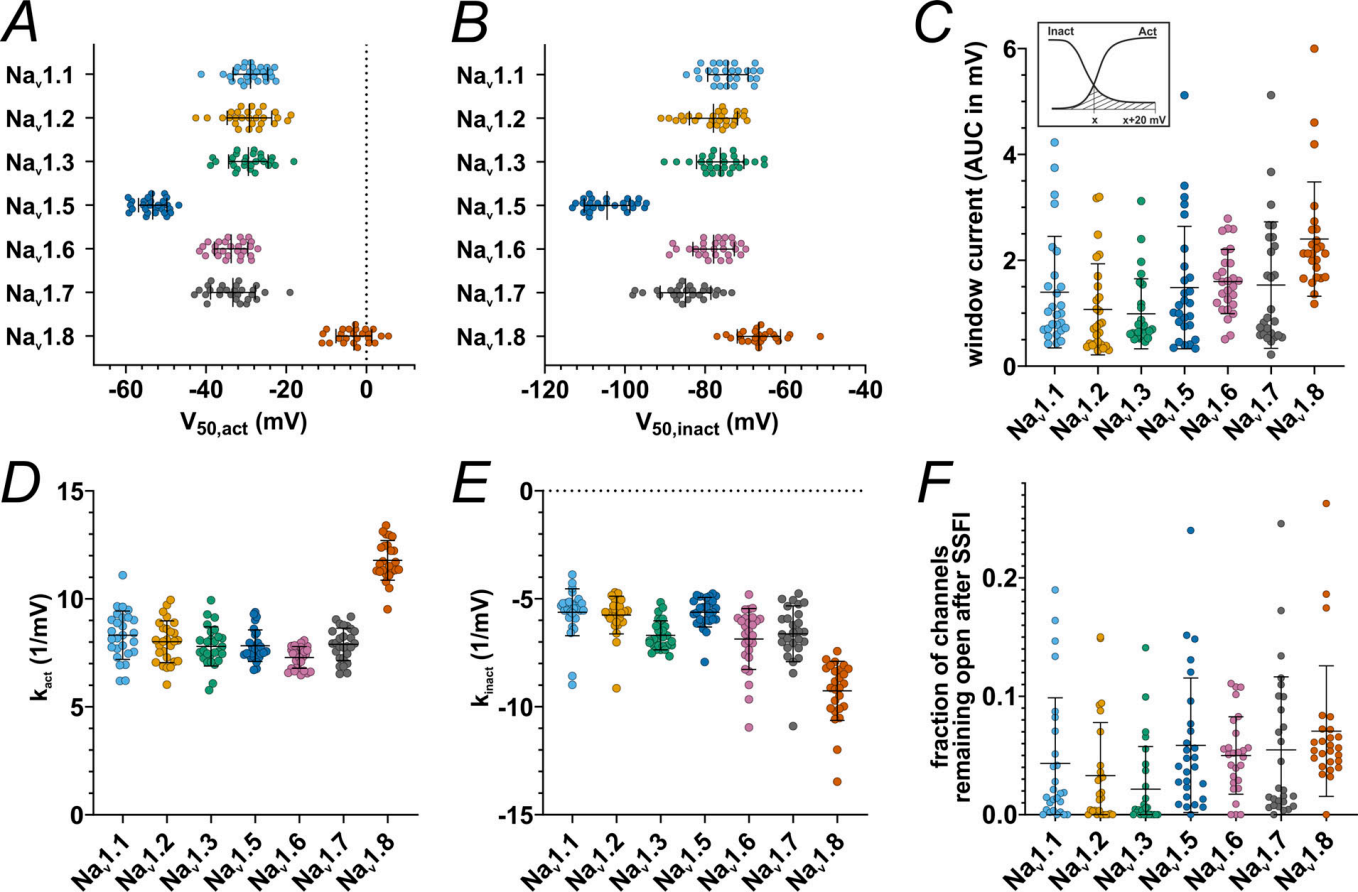
**Activation and SSFI parameters and window current. (A)** *V*_*50*_ values obtained from activation Boltzmann fits. **(B)***V*_*50*_ values obtained from SSFI Boltzmann fits. **(C)** AUC values underneath the superimposed activation and SSFI Boltzmann fit curves, i.e., the window current. Inlet: Schematic depiction of AUC calculation, *x* being the membrane voltage of the activation and SSFI Boltzmann fit curve intersection. **(D)** Slope values *k* obtained from activation Boltzmann fits. **(E)** Slope values *k* gathered from SSFI Boltzmann fits. **(F)** Bottom values gathered from SSFI Boltzmann fits, indicating the fraction of channels remaining open after complete SSFI. Data are shown as mean ± SD. Statistical significance from multiple comparisons has not been indicated in the figure panels for readability purposes but can be consulted in Tables S3, S4, S5, and S6.

To gain a comparable oversight of the biophysics of VGSC isoforms involved in nociception, we examined Na_v_1.1, Na_v_1.2, Na_v_1.3, Na_v_1.5, Na_v_1.6, Na_v_1.7, and Na_v_1.8 for their voltage dependence of activation and SSFI (Fig. 1 and Fig. 2). Of all the channels examined two stood out: Na_v_1.5 activated and fast inactivated at more hyperpolarized potentials than all other VGSC isoforms (Fig. 1 G; and Fig. 2, A and B; and Tables 2 and S3), whereas Na_v_1.8 was set apart by its more depolarized activation and SSFI (Fig. 1 J; and Fig. 2, A and B; and Tables 2 and S3). The TTXs VGSCs examined activated at intermediate voltages, with Na_v_1.6 and Na_v_1.7 being grouped at slightly more hyperpolarized voltages than Na_v_1.1, Na_v_1.2, and Na_v_1.3 (Fig. 1; and Fig. 2, A and B; and Tables 2 and S3). Data obtained from fitting conductance-voltage or current-voltage curves to the Boltzmann equation are summarized in Fig. 2 and Tables 2, S3, S4, S5, and S6. Comparison of the Boltzmann equation fitting results showed differences between the VGSC isoforms in all obtained parameters (P < 0.0001 for all ANOVA or Kruskal–Wallis tests).

**Table 2.** Descriptive statistics of parameters obtained from fitting voltage dependence of activation and SSFI with the Boltzmann equation

	Voltage dependence of activation			Voltage dependence of SSFI				Intersection of activation and SSFI Boltzmann fit curves (mV)	Window current (AUC in mV)	*n*
	*V50*_*act*_ (mV)	*k*_*act*_ (1/mv)	*n*	*V50*_*inact*_ (mV)	*k*_*inact*_ (1/mV)	Fraction of channels remaining open after SSFI (%)	*n*			
Na_v_1.1	−28.9 ± 4.3	8.3 ± 1.1	27	−74.3 ± 5.0	−5.6 ± 1.1	4.3 ± 5.5	27	−56.1 ± 4.6	1.398 ± 1.054	27
Na_v_1.2	−29.2 ± 5.6	8.0 ± 1.0	27	−77.9 ± 6.0	−5.8 ± 0.9	3.3 ± 4.5	27	−57.7 ± 5.6	1.073 ± 0.861	27
Na_v_1.3	−29.5 ± 4.9	7.8 ± 0.9	27	−76.2 ± 5.9	−6.7 ± 0.7	2.1 ± 3.5	27	−54.7 ± 4.3	0.989 ± 0.661	27
Na_v_1.5	−53.3 ± 3.5	7.8 ± 0.7	27	−104.5 ± 5.7	−5.6 ± 0.7	5.9 ± 5.7	27	−83.1 ± 4.6	1.484 ± 1.156	27
Na_v_1.6	−33.8 ± 4.2	7.3 ± 0.5	27	−77.9 ± 5.2	−6.9 ± 1.4	5.0 ± 3.3	27	−56.6 ± 4.7	1.598 ± 0.608	27
Na_v_1.7	−33.3 ± 5.6	7.9 ± 0.8	27	−84.9 ± 6.3	−6.6 ± 1.3	5.5 ± 6.2	27	−61.6 ± 5.6	1.533 ± 1.195	27
Na_v_1.8	−3.1 ± 4.5	11.8 ± 0.9	25	−66.6 ± 5.4	−9.3 ± 1.4	7.1 ± 5.5	26	−38.9 ± 5.1	2.401 ± 1.079	25
Na_v_1.9^a^	−52.3 ± 9.8	11.0 ± 3.2	24	−68.9 ± 10.3	−12.2 ± 4.9	22.4 ± 9.8	17	−61.0 ± 6.3	12.952 ± 4.114	15

Data are shown as mean ± SD.

a Na_v_1.9 Boltzmann values were derived from pooled previously published data from Leipold et al. (2013) and Leipold et al. (2015).

The overlap between activation and SSFI curves is often referred to as window current (Wang et al., 1996; Magistretti and Alonso, 1999; Abriel et al., 2001; Osteen et al., 2017). The intersection of activation and SSFI curves (i.e., the peak of the window current) of Na_v_1.7 was ∼5 mV more hyperpolarized than that for all other TTXs channels tested except for Na_v_1.2 (Fig. 1 and Tables 2and S5). This indicates that in this heterologous expression system, a higher fraction of Na_v_1.7 channels are in an open state (i.e., activated and not yet fast inactivated) at more hyperpolarized voltages than the other assumed subthreshold channels, Na_v_1.1, Na_v_1.3, and Na_v_1.6, and thus could contribute to cell depolarization at subthreshold voltages.

Window current size was larger for Na_v_1.8 than for every other VGSC examined except for Na_v_1.6. Among TTXs channel isoforms, Na_v_1.6 elicited slightly higher window currents than Na_v_1.2 and Na_v_1.3 but did not differ from Na_v_1.1 and Na_v_1.7 (Fig. 2 C and Tables 2 and S5), underlining their equal potential to support subthreshold depolarization. Concerning the fraction of channels remaining open after SSFI, we observed a trend for Na_v_1.2/3 and Na_v_1.8 to smaller or larger currents, respectively (Fig. 2 F and Tables 2 and S6).

**Figure 3.**
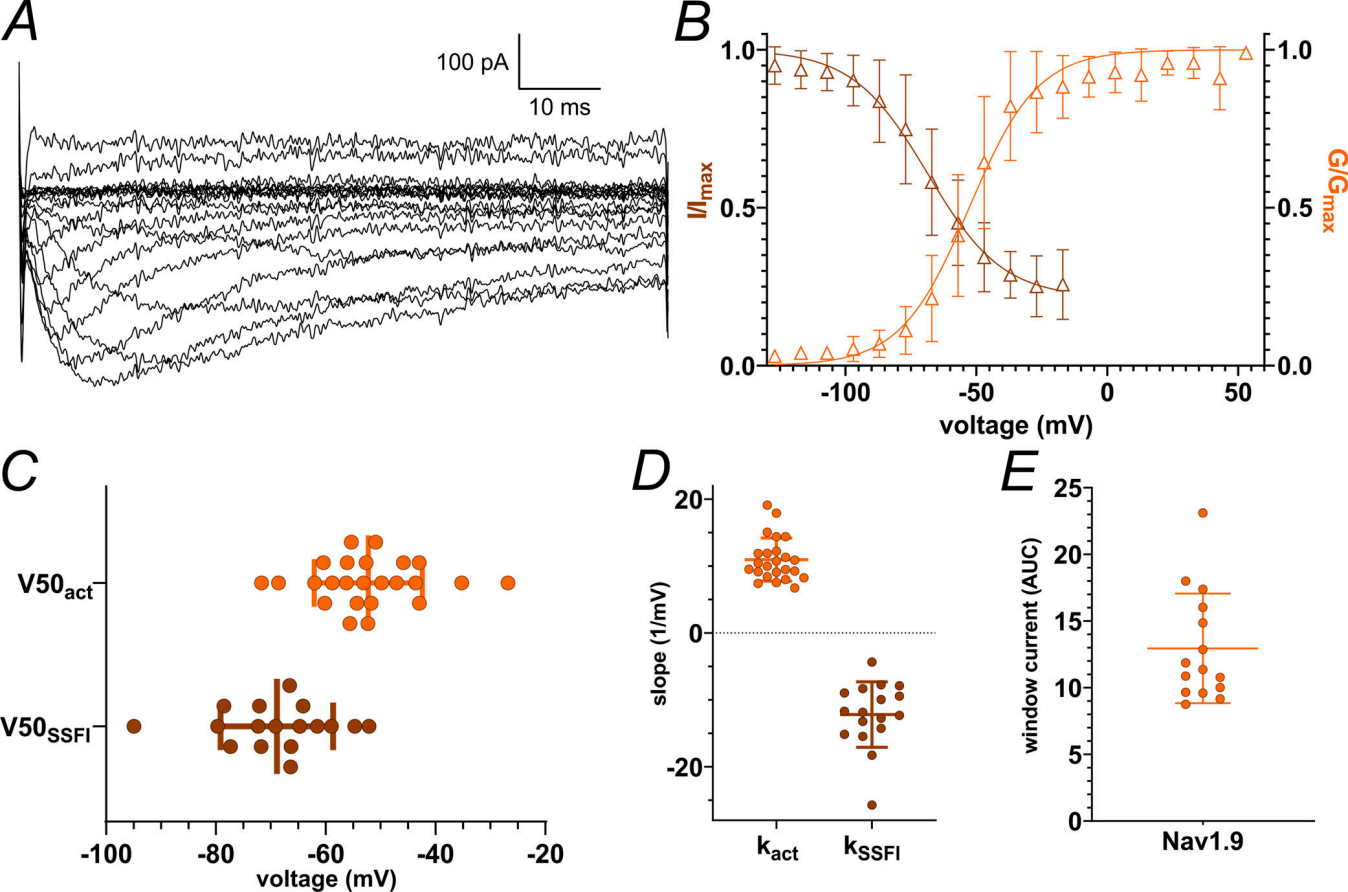
**Summary of Na**_**v**_ **1.9 activation and SSFI data.** Data from Leipold et al. (2013), Leipold et al. (2015) were pooled and analyzed concerning Na_v_1.9 gating and window currents. **(A)** Example current traces of Na_v_1.9 elicited by the activation protocol. Note that cells were stimulated with a −127 to + 63 mV voltage range. **(B)** Overlay of activation conductance-voltage and SSFI current-voltage curves. **(C)***V*_*50*_ values obtained from activation and SSFI Boltzmann fits. **(D)** Slope values *k* obtained from activation and SSFI Boltzmann fits. **(E)** AUC values underneath the superimposed activation and SSFI Boltzmann fit curves, i.e., the window current. Data are shown as mean ± SD.

To complete our data set with the last remaining VGSC essential for nociceptors, we analyzed pooled Na_v_1.9 data from previously published recordings (Leipold et al., 2013, 2015) (Fig. 3 and Table 2). While activation of Na_v_1.9 occurred at hyperpolarized voltages comparable with Na_v_1.5, SSFI voltage dependence resembled the depolarized inactivation of Na_v_1.8 (Fig. 3, B and C; and Table 2). Na_v_1.9 displayed pronounced persistent currents (Fig. 3 A) as described previously (Herzog et al., 2001; Maingret et al., 2008). These properties enable the formation of major window currents exceeding those of all other VGSC isoforms measured in this study (Fig. 3 E and Table 2).

### Ramp and window currents of only some VGSC isoforms may arise from a similar mechanism

**Figure 4.**
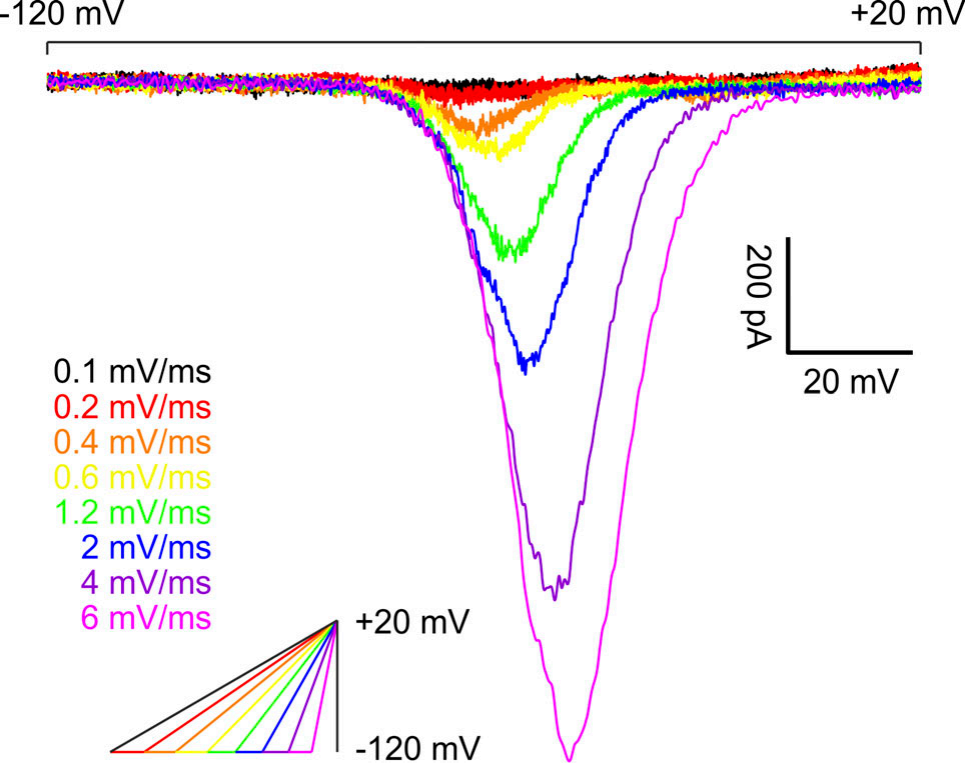
**Example traces of Na**_**v**_ **1.7 ramp currents elicited by slowly depolarizing ramp stimuli.** Voltage protocol depicted in inlet. Scale bars apply to current traces only.

Ramp stimuli evoked a bell-shaped inward current response for all tested VGSC isoforms (Fig. 4), as reported previously (Cummins et al., 1998; Abriel et al., 2001; Herzog et al., 2003; Power et al., 2012; Estacion and Waxman, 2013; DeCaen et al., 2014; Han et al., 2015a; Zhang et al., 2017). For cells expressing Na_v_1.3, we observed two-peaked ramp currents for slowly depolarizing ramps (i.e., 0.1 mV/ms and partly 0.2 mV/ms) in six out of a total of 27 recordings, similar to what was reported previously (Estacion and Waxman, 2013) (see Fig. S1). For the remaining cells and higher ramp rates, a delayed current decay was observed, potentially corresponding to Na_v_1.3 persistent current.

**Figure 5.**
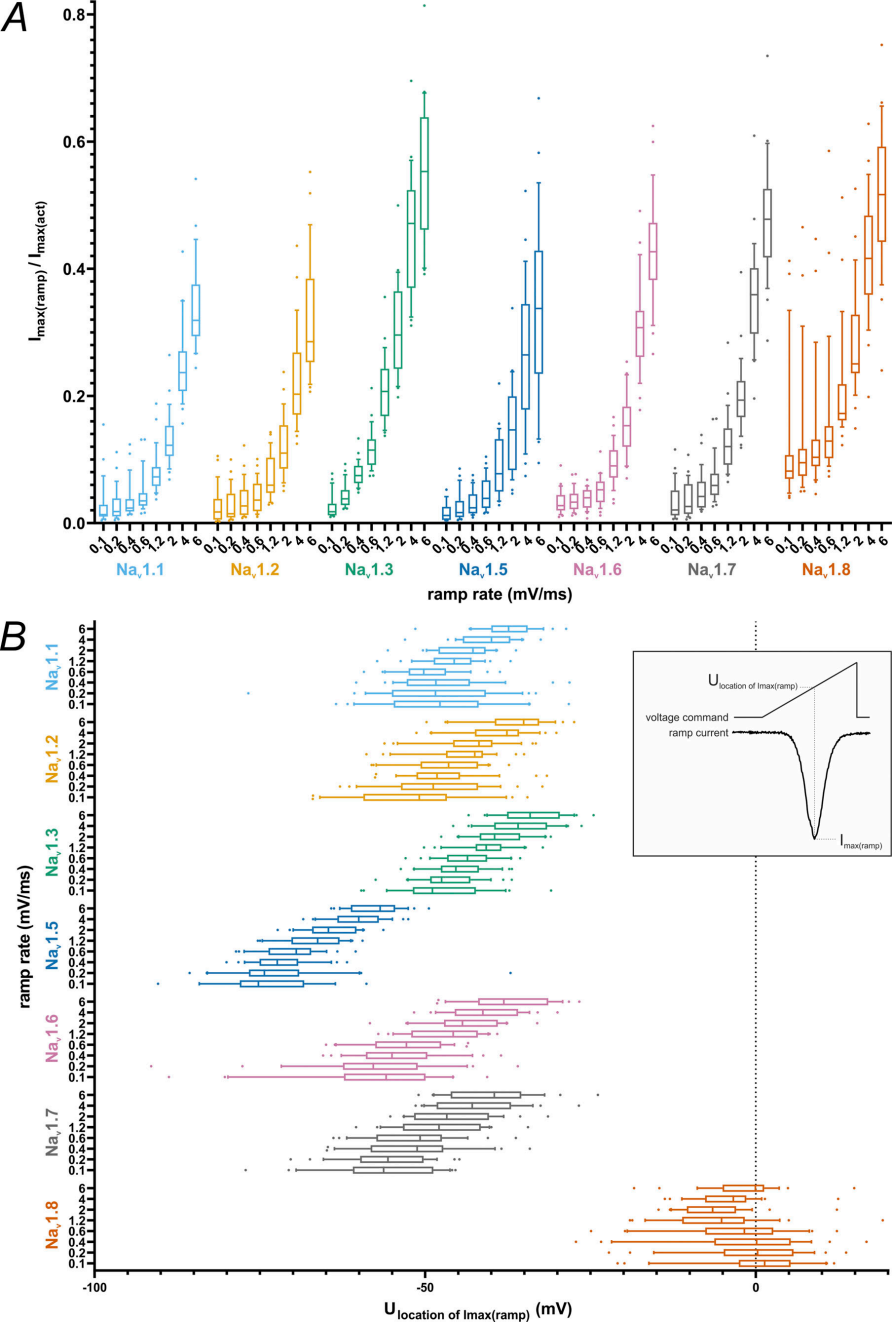
**Current-voltage parameters obtained from ramp current measurements. (A)** Maximum inward current from ramp current measurement normalized to maximum inward current from activation measurements. **(B)** Voltage at which the maximum inward current occurred during ramp current measurement. Data are shown as box plots with whiskers indicating the 10th and 90th percentile and dots for measurements below the 10th and above the 90th percentile.

Ramp currents for all tested isoforms increased with steeper ramp stimuli, most likely due to more channels activating before the onset of fast inactivation. As determined in post hoc testing, for the lowest ramp rates (i.e., ≤0.4 mV/ms), most VGSC isoforms did not differ in maximum inward ramp current; only Na_v_1.8 elicited higher maximum ramp currents than the other VGSC isoforms (Table S7). For medium ramp rates (0.6–1.2 mV/ms), Na_v_1.3 and Na_v_1.8 exceed the other VGSC isoforms, whereas for the highest ramp rates (2–6 mV/ms), Na_v_1.6 and Na_v_1.7 gradually catch up, leaving Na_v_1.1, Na_v_1.2, and Na_v_1.5 grouped at lower values (Fig. 5 A and Tables S7 and S8).

We observed that the maximum inward current evoked by ramp stimulation was affected more by the ramp rate than by the VGSC isoform: the difference between VGSC isoforms (*F*[6, 1429] = 153.0, P < 0.0001) and ramp rate (*F*[7, 1,429] = 974.8, P < 0.0001) made up for 9.7% and 71.8% of total variation, respectively (Fig. 5 A and Table S7). Interaction between these terms accounted for 3.3% of total variation (*F*[42, 1,429] = 7.528, P < 0.0001).

Ancillary to the size of the maximum inward ramp current, we saw differences in its voltage dependence (Fig. 5 B and Table S7) between VGSC subtypes (*F*[6, 1,429] = 1,748, P < 0.0001; 82.5% of total variation), ramp rates (*F*[7, 1,429] = 90.15, P < 0.0001; 5% of total variation), and their interaction (*F*[42, 1,429] = 5.17, P < 0.0001; 1.7% of total variation). Using multiple comparison testing, VGSC isoforms showed a distribution matching the results of activation/SSFI *V*_*50*_ results and Boltzmann fit intersections for the vast majority, with Na_v_1.5 and Na_v_1.8 ramp currents occurring at more hyperpolarized and depolarized voltages, respectively, than all other TTXs ramp currents tested (Tables S7 and S9). TTXs channels are grouped at intermediate values, with Na_v_1.6 and Na_v_1.7 shifted toward more hyperpolarized values than Na_v_1.1, Na_v_1.2, and Na_v_1.3. For steeper ramps, this effect intensified, especially for Na_v_1.7 and Na_v_1.3.

**Figure 6.**
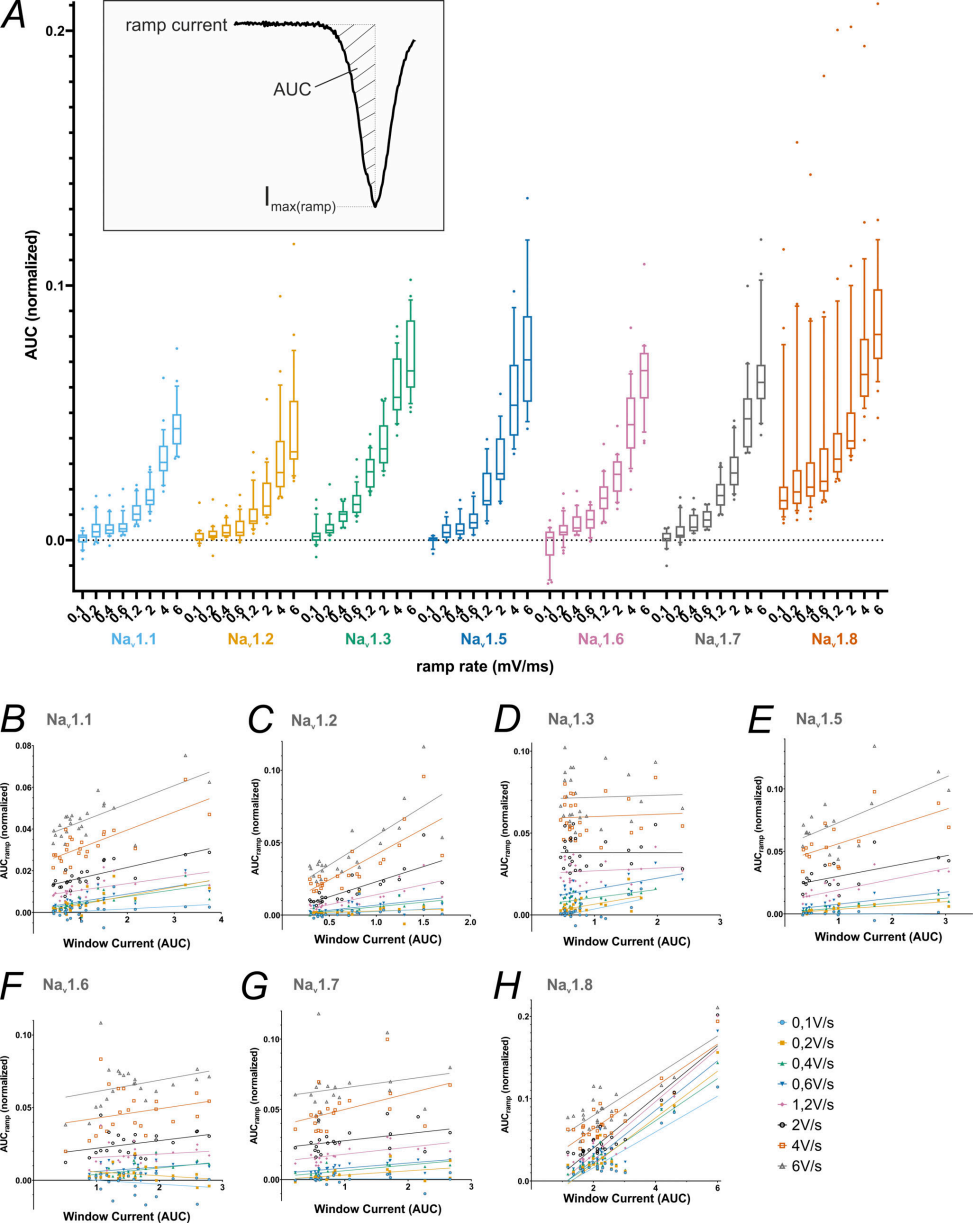
**AUC values from ramp current measurements correlate with window current measurements for some VGSC isoforms. (A)** AUC values obtained from ramp current measurements, normalized to the maximum inward current from activation measurements for each cell (inlet depicts a schematic example of an AUC underneath a ramp current curve. Note that the AUC was calculated only up to the point of maximum inward current to make up for Na_v_1.8 ramp currents being cut off at +20 mV). Data are shown as box plots with whiskers indicating the 10th and 90th percentile. **(B–H)** Correlation between AUC values from ramp current measurements and window current values from activation/SSFI measurements. Data are shown as AUC values plotted against the window current values of the respective cell with a linear regression line for each ramp rate.

As a measure of charge added to a cell during slow depolarizations such as the subthreshold phase, we investigated the AUC underneath ramp currents up to the ramp current peak (Fig. 6 A and Table S7). Post hoc testing evidenced that Na_v_1.8 had larger AUC values than the other VGSC isoforms for all ramp rates (Table S10). No distinct differences in the AUC between the remaining channel isoforms were observed for lower ramp rates (≤0.6 mV/ms). With rising ramp rates, the AUC of Na_v_1.3, Na_v_1.5, Na_v_1.6, and Na_v_1.7 gradually surpasses the AUC of Na_v_1.1 and Na_v_1.2. The AUC is affected by the VGSC isoform (*F*[6, 1180] = 95.75, P < 0.0001; 15% of total variation), ramp rate (*F*[7, 1180] = 281.6, P < 0.0001; 51.4% of total variation), and their interaction (*F*[42, 1180] = 1.984, P = 0.0002; 2.2% of total variation).

When analyzing the AUC under the entire ramp current curve for all VGSC examined except for Nav1.8, AUC size distribution among the channels was similar (Fig. S2 and Table S11). The influence of the ramp rate on the AUC results was more pronounced when analyzing the full ramp current curve (F[7, 978] = 449.5, P < 0.0001; 68.1% of total variation), while the impact of the VGSC isoform was decreased (F[5, 978] = 49.56, P < 0.0001; 5.4% of total variation) as was their interaction (F[35, 978] = 5.485, P < 0.0001; 4.2% of total variation).

Some authors refer to window current as corresponding to persistent or ramp current (Blair and Bean, 2002; Enomoto et al., 2006; Matsutomi et al., 2006; Vasylyev and Waxman, 2012; Estacion and Waxman, 2013), although so far we are unaware of a study investigating this potential link. We used Spearman’s rank correlation to test for a relationship between window current and ramp current AUC (Fig. 6, B–H, and Table S12). For Na_v_1.1 and Na_v_1.2, a positive correlation was identified for all ramp rates. While Na_v_1.5, Na_v_1.7, and Na_v_1.8 exhibited a positive correlation for most, but not all, ramp rates, for Na_v_1.3 and Na_v_1.6, a positive correlation was detected for only one ramp rate. Note that smaller Na_v_1.5 sample sizes due to the established exclusion criteria might limit its correlation accuracy. Our results indicate that ramp and window currents correlate only in specific cases and are thus likely to be generated by distinct mechanisms.

### All tested VGSCs activate during the AP subthreshold phase except for Na_v_1.8

**Figure 7.**
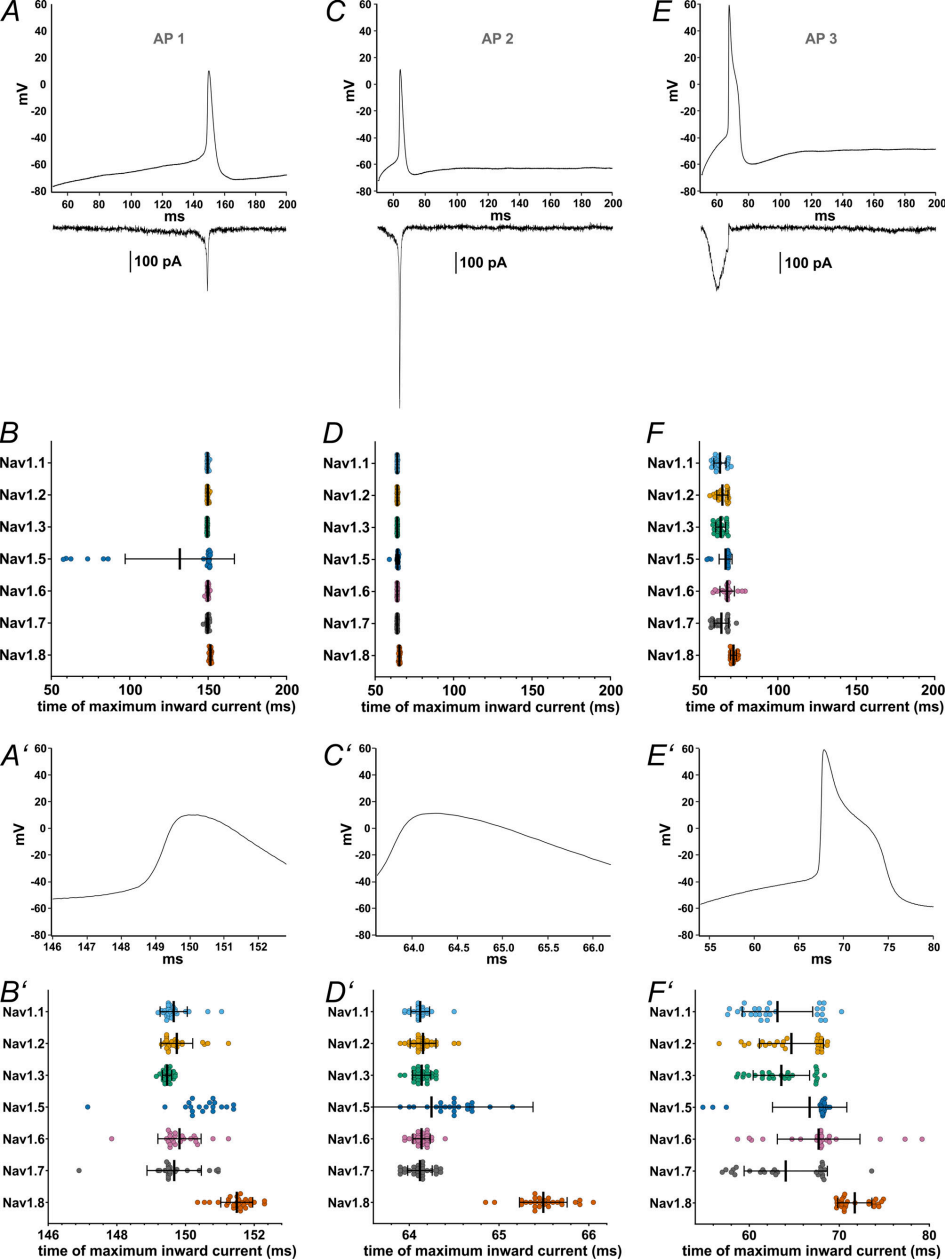
**Maximum inward current occurs at different time points during AP clamping for each VGSC isoform. (A,** **C, and E)** AP waveforms (AP1–AP3, respectively) recorded from iPSC-derived nociceptors used as voltage commands in AP clamping, with representative current traces, all from the same Na_v_1.7 cell, charted underneath. **(B, D, and F)** Time point at which the maximum inward current during the AP (AP1–AP3, respectively) occured. **(A′–F′)** Zoomed in depictions of figures A–F (excluding the current example traces). Data are shown as mean ± SD. Statistical significance from multiple comparisons has not been indicated in the figure panels for readability purposes but can be consulted in Table S14.

We used single AP recordings from human iPSC-derived nociceptors (control cells from Meents et al. [2019]) as a voltage command to determine the contribution of each VGSC to the phases of the AP. The three pre-recorded APs (AP1, AP2, and AP3) differ in their subthreshold properties, their width, and size of the overshoot (Fig. 7, A, C, and E; and Table 1).

**Figure 8.**
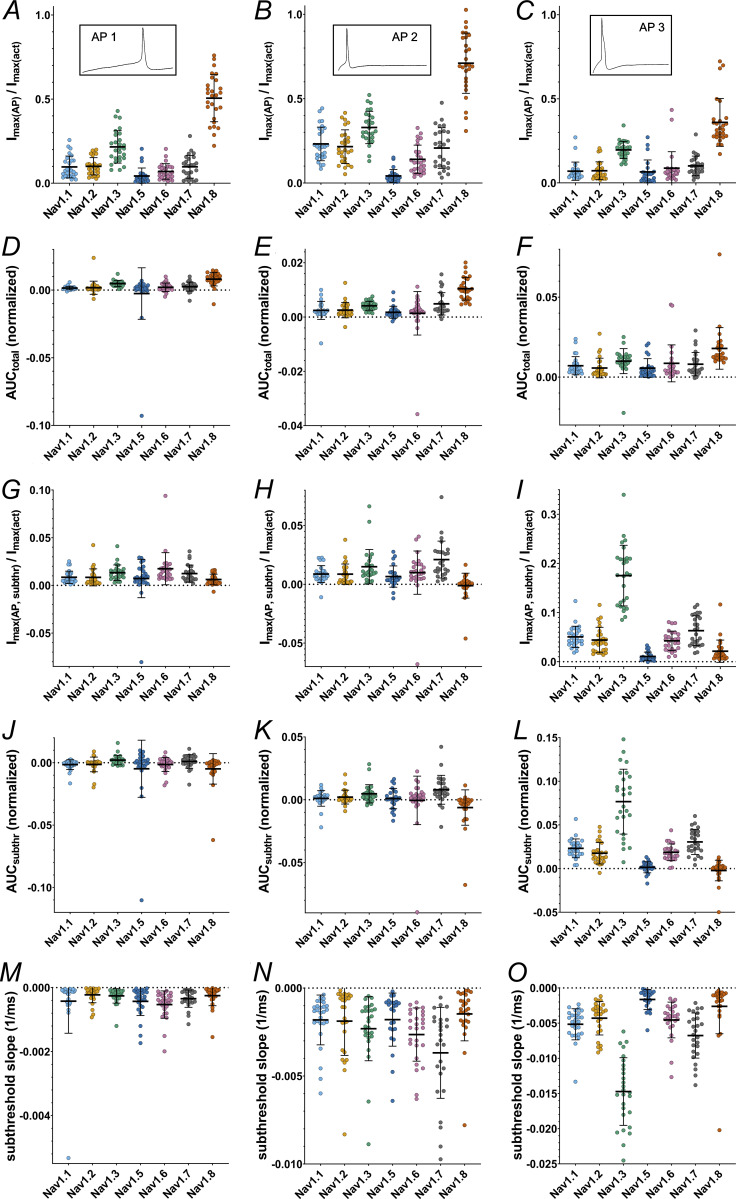
**Current and AUC parameters obtained from AP clamping. (A–C)** Maximum inward current during the total command AP (AP1–AP3, respectively) (inlets indicate the AP command). **(D–F)** Area under the current response curve to the total command AP (AP1–AP3, respectively). **(G–I)** Maximum inward current during the subthreshold phase of the command AP (AP1–AP3, respectively). **(J–L)** Area under the current response curve to the subthreshold phase of the command AP (AP1–AP3, respectively). **(M–O)** Slope of the inward current measured during the subthreshold phase of the command AP (AP1–AP3, respectively). Data are normalized to the maximum inward current of activation measurement of the correspondent cell and shown as mean ± SD. Statistical significance from multiple comparisons has not been indicated in the figure panels for readability purposes but can be consulted in Table S14.

The time point during the AP at which the maximum inward current is elicited differed between certain VGSC isoforms for all AP commands (Fig. 7, B, D, and F; and Tables S13 and S14; P < 0.0001 for all Kruskal–Wallis tests), as did the maximum inward current sizes (Fig. 8, A–C and Table S13, P < 0.0001 for all Kruskal–Wallis tests) and the area under the current response curve (Fig. 8, D–F and Table S13; P < 0.0001 for all Kruskal–Wallis tests).

Multiple comparisons revealed that Na_v_1.8 currents were elicited at later time points than all other tested VGSC isoforms except for Na_v_1.5, namely, after the AP peak (Table S14). Na_v_1.5 currents occurred slightly later within the AP time course as well, whereas Na_v_1.3 leaned toward earlier time points in the subthreshold phase (Fig. 7, B′, D′, and F′; and Tables S13 and S14). Current sizes and AUC sizes were similarly distributed between the AP commands, with Na_v_1.8 and Na_v_1.3 exhibiting larger and Na_v_1.5 exhibiting slightly lower values for maximum inward current (Fig. 8, A–F, and Tables S13 and S14).

The examination of the time point of maximum inward current during AP3 stood out since some VGSC isoforms displayed results with a bimodal distribution (Fig. 7 F′). Post hoc data revision revealed a transient current peak during the upstroke of the AP command in varying degrees for Na_v_1.1–1.3 and Na_v_1.7, most likely caused by insufficient pipette capacitance compensation. When this transient current exceeded the more or less evenly pronounced subthreshold influx, the measured time point was shifted, forming the second cluster depicted in Fig. 7 F′. For Na_v_1.8, which also displayed a slightly clustered distribution, no similar effect could be observed. By excluding the measurements impeded by above-mentioned transients, channel distribution became even more distinct: Na_v_1.1–1.3 and Na_v_1.7 had their maximum influx during the subthreshold phase of the AP command, considerably before Na_v_1.5, Na_v_1.6, and Na_v_1.8 (Fig. S3 and Table S14).

During the subthreshold phase of the APs maximum inward current, AUC and inward current slope differed between the VGSC isoforms (P < 0.0001 for all Kruskal–Wallis tests except for AP1 subthreshold AUC [P = 0.0008]). On average, maximum inward currents increased with increasing subthreshold slope of the AP commands (Fig. 8, G–I and Table S13). Post-hoc testing indicates that Na_v_1.8 and Na_v_1.5 elicited little to no current during the subthreshold phase, while maximum inward current was higher for Na_v_1.3 (AP1 and AP3), Na_v_1.7 (AP2 and AP3), and, for AP1, Na_v_1.6 (Fig. 8, G–I and Table S14). Especially for AP3 with the steepest subthreshold slope, Na_v_1.3 currents increased drastically (Fig. 8 I and Tables S13 and S14). The remaining TTXs isoforms had intermediate maximum inward current responses.

A similar pattern was observed for the subthreshold phase AUC (Fig. 8, J–L, and Tables S13 and S14). Na_v_1.5 and Na_v_1.8 AUC values remained close to zero for all AP commands (note that negative AUC values occur due to undulation of the current base rate and/or drift). Na_v_1.7 and Na_v_1.3 displayed larger subthreshold AUC values (the latter particularly for AP3), thereby adding a larger charge to a cell during subthreshold depolarization and potentially alleviating initiation of an AP.

As another measure of channel contribution to subthreshold depolarization, we determined the slope of currents elicited during the subthreshold phase of AP clamping by each VGSC isoform (Fig. 8, M–O, and Tables S13 and S14). Steeper subthreshold slopes were documented for Na_v_1.7 (AP2 and AP3), Na_v_1.6 (AP1 and AP2), and, with emphasis on AP3, Na_v_1.3 compared with the other tested channels. In contrast, Na_v_1.5 and Na_v_1.8 subthreshold slopes were minor. The data are in line with the assumption that Na_v_1.8 is mainly activated during the upstroke of a nociceptor AP, while TTXs channels (predominantly Na_v_1.7, Na_v_1.6, and—especially for steeper subthreshold depolarizations—Na_v_1.3) contribute in earlier, subthreshold phases.

### In silico simulation suggests significant contribution of Na_v_1.9 to the fast upstroke of the AP and its shoulder

With the large data set in hand, which contains patch-clamp recordings of all VGSCs relevant in nociceptors, we now aimed to obtain insights into the contribution of each isoform to the AP by in silico experiments. Our computational model comprises the sodium currents of Na_v_1.1–Na_v_1.3, Na_v_1.5–Na_v_1.8 (derived from the current trace measurements of this study), and Na_v_1.9 (derived from current trace measurements of Leipold et al. [2013] and Leipold et al. [2015]), and a generic potassium and leak current (see Materials and methods and Online supplemental material).

To generate an adequate fit of the recorded current traces of the different VGSCs, we implemented a modification to the original Hodgkin–Huxley model: we introduced a waiting state after pulse onset due to which the fast inactivation gate (denoted as *h* in the Hodgkin–Huxley framework) is delayed (see Materials and methods and Online supplemental material). This waiting state could mimic the time during which VGSC may open and are not ready to inactivate yet. This modification led to improved trace fitting for Na_v_1.1–1.3 and Na_v_1.5–1.8, as can be seen in the reduced deviation from the original trace (see Online supplemental material). The Hodgkin–Huxley–like models of each sodium channel subtype were integrated into a nonspatial model of an Aδ-fiber and a CMi, which were used to simulate APs in response to current injections (see Materials and methods and Online supplemental material).

**Figure 9.**
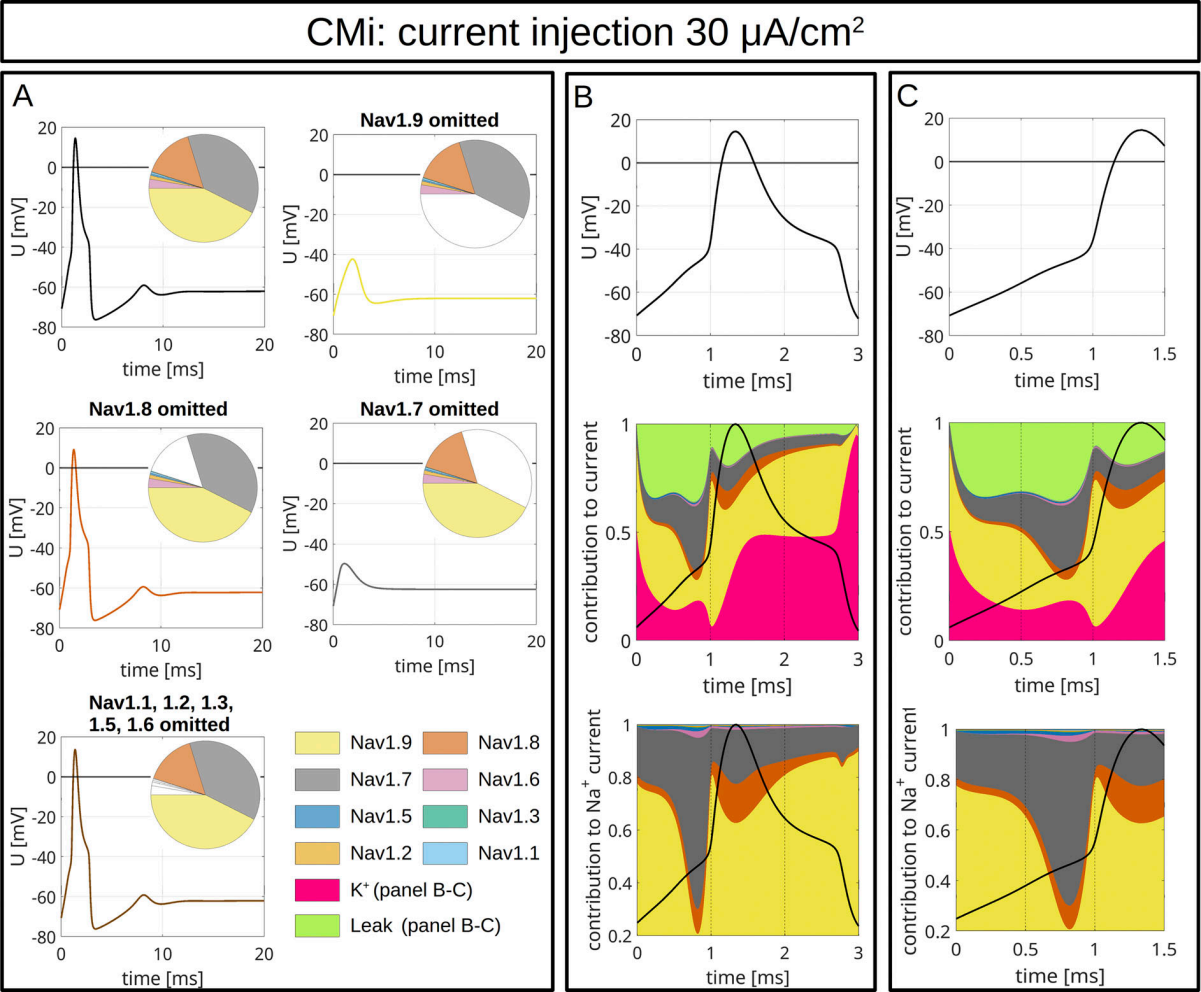
**Computer simulations of APs in a CMi.** The pie charts indicate the abundance of different VGSC isoforms. APs are triggered by an injection of 30 μA/cm^2^, which persists until the end of the simulation. **(A)** The upper left panel shows the AP for the relative abundance of VGSC isoforms quantified in spatial gene expression. The initial condition of the simulation is the resting state of the fiber. The other panels show changes of the AP resulting from removal of Na_v_1.9 (upper right), Na_v_1.8 (middle left), Na_v_1.7 (middle right), and Na_v_1.1–1.3, 1.5, and 1.6 (bottom left). The VGSC isoforms are coded by the colors specified in the bottom right panel. **(B and C)** VGSC isoform contributions to the simulated AP depicted in the upper left panel of A. The upper panels of B and C show the membrane potential during the initial 3 ms (B) and 1.5 ms (C) of the simulated AP. The middle panels show the relative contribution of the different sodium currents, the potassium current, and the leak current over the course of the AP, plotted as stacked individual currents normalized to total current at each time point. The bottom panels show VGSC isoform contributions to the total sodium current, plotted as stacked individual VGSC isoform currents normalized to the total sodium current at each time point. The black line visualizes the shape of the AP. The color coding is identical to that in A.

**Figure 10.**
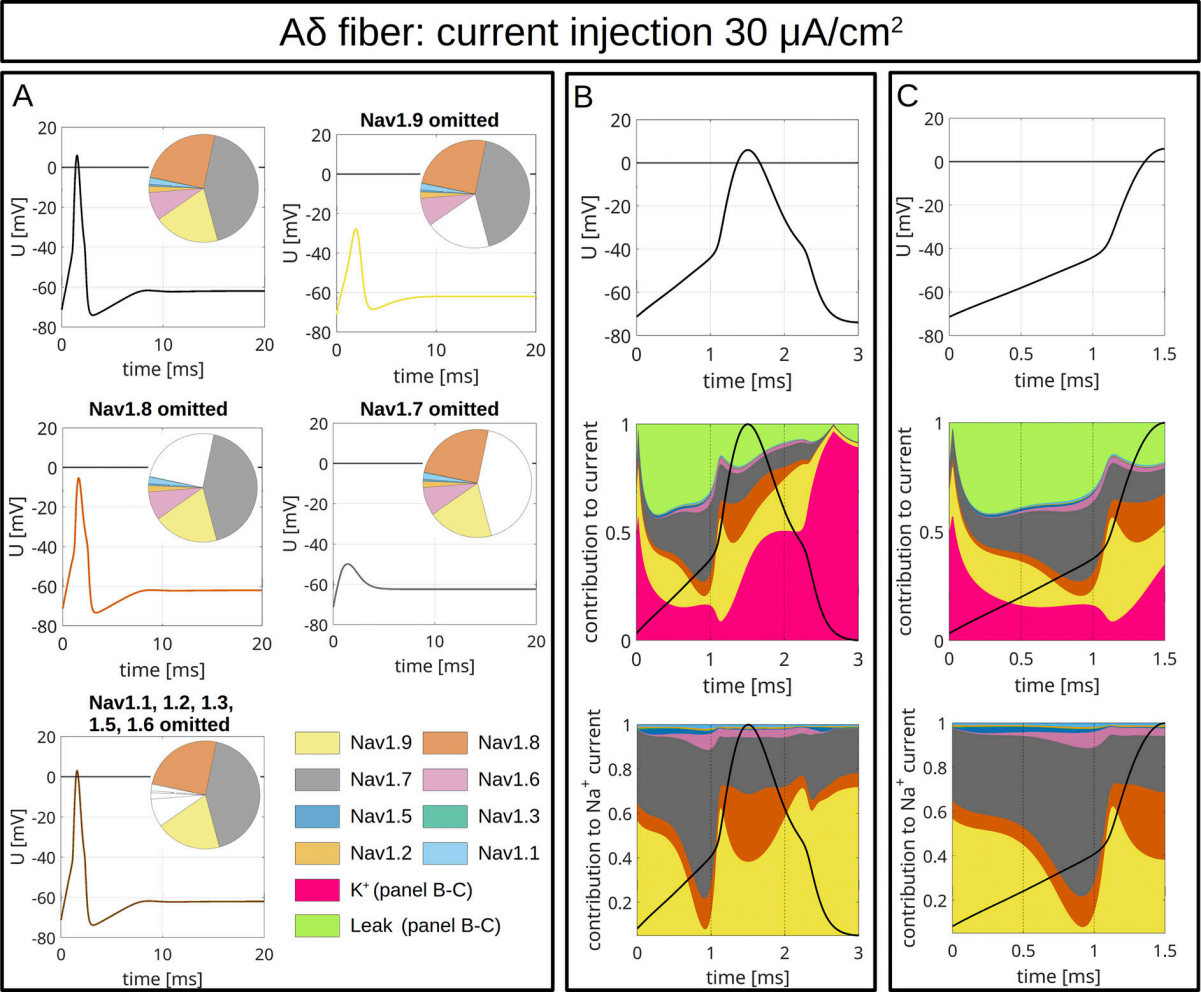
**Computer simulations of APs in an Aδ-fiber.** The pie charts indicate the abundance of different VGSC isoforms. APs are triggered by an injection of 30 μA/cm^2^ which persists until the end of the simulation. **(A)** The upper left panel shows the AP for the relative abundance of VGSC isoforms quantified in spatial gene expression. The initial condition of the simulation is the resting state of the fiber. The other panels show changes of the AP resulting from removal of Na_v_1.9 (upper right), Na_v_1.8 (middle left), Na_v_1.7 (middle right), and Na_v_1.1–1.3, 1.5, and 1.6 (bottom left). The VGSC isoforms are coded by the colors specified in the bottom right panel. **(B and C)** VGSC isoform contributions to the simulated AP depicted in the upper left panel of A. The upper panels of B and C show the membrane potential during the initial 3 ms (B) and 1.5 ms (C) of the simulated AP. The middle panels show the relative contribution of the different sodium currents, the potassium current, and the leak current over the course of the AP, plotted as stacked individual currents normalized to total current at each time point. The bottom panels show VGSC isoform contributions to the total sodium current, plotted as stacked individual VGSC isoform currents normalized to the total sodium current at each time point. The black line visualizes the shape of the AP. The color coding is identical to that in A.

To model CMi (Fig. 9 and Fig. S4) and Aδ-fibers (Fig. 10 and Fig. S5), we accounted for the fiber-specific expression patterns of VGSCs quantified in Tavares-Ferreira et al. (2022), as these sequencing results provide well-accessible, reliable human expression data of all VGSCs in sensory neurons.

The maximal total voltage-gated sodium, potassium, and leak conductivities were chosen to recapitulate the experimentally observed AP morphology. In the simulations, the APs were triggered by current injection of 30 µA/cm^2^ (Fig. 9 and Fig. 10) or 40 µA/cm^2^ (Fig. S4 and Fig. S5) throughout the experiment. Both fiber types revealed an AP with an overshoot and a shoulder (stronger for CMi) upon stimulation with 30 µA/cm^2^ (Fig. 9 A and Fig. 10 A) and 40 µA/cm^2^ (Fig. S4 A and Fig. S5 A).

To assess the role of a specific VGSC isoform, we removed it from the model and observed the resulting AP changes (Fig. 9 A, Fig. 10 A, Fig. S4 A, Fig. S5 A, and Fig. S6). Notably, the removal of an isoform resulted in a reduction of the total *G*_*Na*_.

The most striking finding of our simulations is the strong contribution of Na_v_1.9 to the fast upstroke of the AP as well as to the formation of a shoulder in both fiber types (Fig. 9, B and C; Fig. 10, B and C; Fig. S4, B and C; Fig. S5, B and C; Fig. S6; and Fig. S7). Omission of Na_v_1.9 at low stimulation intensities aborted AP generation, very similar to omission of Na_v_1.7 (Fig. 9 A and Fig. 10 A). The latter simulates loss-of-function as it may occur for Na_v_1.7 in patients suffering from chronic insensitivity to pain (Lampert et al., 2014). While removal of Na_v_1.8 from the model resulted in AP waveforms that failed to overshoot, this intervention did not affect formation of the AP shoulder, suggesting that other subtypes such as Na_v_1.9 may contribute to shoulder formation (Fig. 9 A and Fig. 10 A).

To investigate this further, we reduced the conductance of Na_v_1.9 gradually in our CMi-model while increasing that of the other sodium channels to keep the overall VGSC conductance constant (Fig. S7). Reducing Na_v_1.9 gradually flattened the shoulder; the fast upstroke of the AP turned less steep, and subthreshold depolarizations following the AP disappeared. Additionally, the after hyperpolarization was reduced, which may reflect a weaker activation of voltage-gated potassium channels due to the vanishing of the shoulder. Removal of Na_v_1.1–1.3, Na_v_1.5, and Na_v_1.6 (at the same time) had no significant impact on the AP of the CMi but reduced the overshoot in the Aδ-fiber (Fig. 9 A, Fig. 10 A, Fig. S4 A, Fig. S5 A, and Fig. S6).

Fig. 9, B and C; and Fig. 10, B and C, show the relative contributions of the different VGSC isoforms to the simulated APs of the CMi and the Aδ-fiber, respectively. In both fiber types, the relative contribution of Na_v_1.7 is maximal right before or at the beginning of the fast upstroke of the AP (Fig. 9 C, Fig. 10 C, Fig. S4 C, and Fig. S5 C), in line with reports on Na_v_1.7 as a subthreshold or threshold channel (Rush et al., 2007; Vasylyev and Waxman, 2012; Meents et al., 2019). The largest relative (Fig. 9, B and C; and Fig. 10, B and C), but also absolute (Fig. S8), conduction by far in our two models is mediated by Na_v_1.9. Most of its relative contribution occurs during subthreshold depolarizations, then during the fast upstroke of the AP (relative and absolute), and, with a prominent second peak (relative and absolute), during the falling phase of the AP coinciding with the shoulder.

Na_v_1.8, on the other hand, shows the largest conductivity during the upstroke, the peak, and the falling phase of the AP (Fig. 9, B and C; Fig. 10, B and C; Fig. S4, B and C; Fig. S5, B and C; and Fig. S8), but when the AP shoulder occurs, it seems to be already partially inactivated. The relative and absolute contribution of Na_v_1.8 and Na_v_1.7 to the AP, especially during the peak, seems to be flipped between the two fiber models investigated here: while in the CMi model, Na_v_1.7 contributes more, it is Na_v_1.8 in the Aδ-fiber model. The other channels show only minor contributions during the course of the AP: Na_v_1.5 shows some activity during subthreshold depolarizations and a small peak during the shoulder in the Aδ-fiber model, while Na_v_1.6 is active at the AP threshold in both models. Na_v_1.6 is continuously active throughout the AP in the Aδ model (Fig. 9, B and C; Fig. 10, B and C; Fig. S4, B and C; Fig. S5, B and C; and Fig. S8).

Gain-of-function mutations of Na_v_1.7 linked to inherited pain syndromes often modify channel gating such that during an AP more channels are activated. Erythromelalgia mutations in Na_v_1.7 lead to hyperpolarized activation of the channel, inducing hyperexcitability when overexpressed in rodent sensory neurons (Körner and Lampert, 2020) and enhancing spontaneous activity in human stem cell–derived nociceptors (Mis et al., 2019). We used our computational model to simulate gain-of-function in two ways: (1) by increasing the maximal conductance of Na_v_1.7 and comparing it to an increased Na_v_1.8 conductance and (2) by shifting activation of Na_v_1.7 to more hyperpolarized potentials.

**Figure 11.**
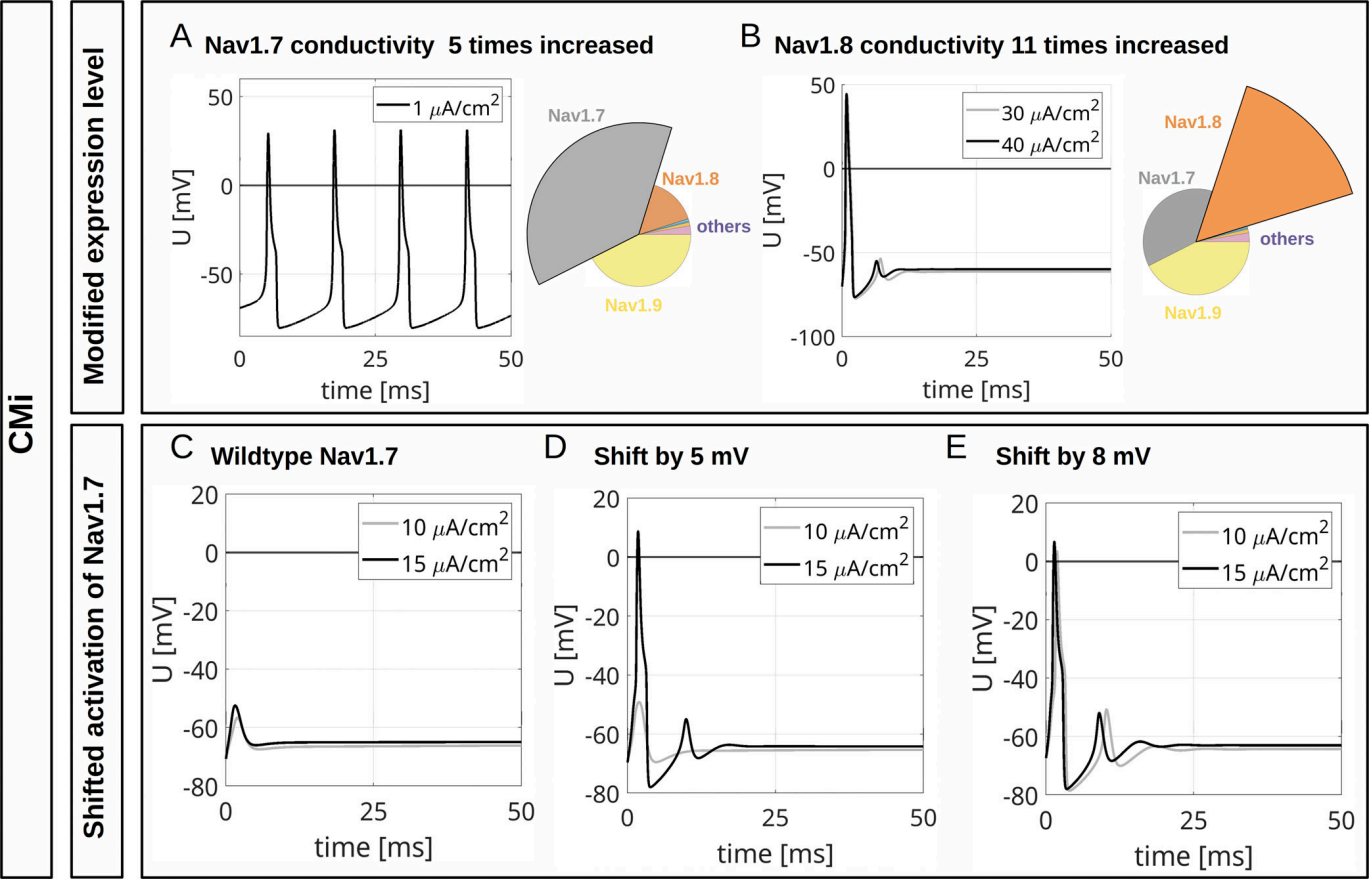
**Computational disease modelling in a CMi. (A)** Repetitive depolarizations resulting from increasing the maximal conductance for Na_v_1.7 to 500% of the WT value. The current injection of 1 μA/cm^2^ persists until the end of the simulation. **(B)** An increase of the maximal conductance for Na_v_1.8 to 1,100% of the WT value results in an isolated AP. The total *G*_*Na*_ in A and B are approximately the same. The current injections of 30 and 40 μA/cm^2^ persist until the end of the simulations. The pie charts indicate the abundance of different VGSC isoforms. **(C)** Current injections of 10 and 15 μA/cm^2^ trigger no APs in the simulated WT CMi (isoform abundances as in Fig. 9 A, upper left panel). **(D)** If the activation of Na_v_1.7 is shifted by 5 mV to hyperpolarized potentials, a current injection of 15 μA/cm^2^ but not of 10 μA/cm^2^ triggers an AP. **(E)** If the activation of Na_v_1.7 is shifted by 8 mV to hyperpolarized potentials, current injections of 10 and 15 μA/cm^2^ trigger an AP. The current injections in C–E persist until the end of the simulation.

**Figure 12.**
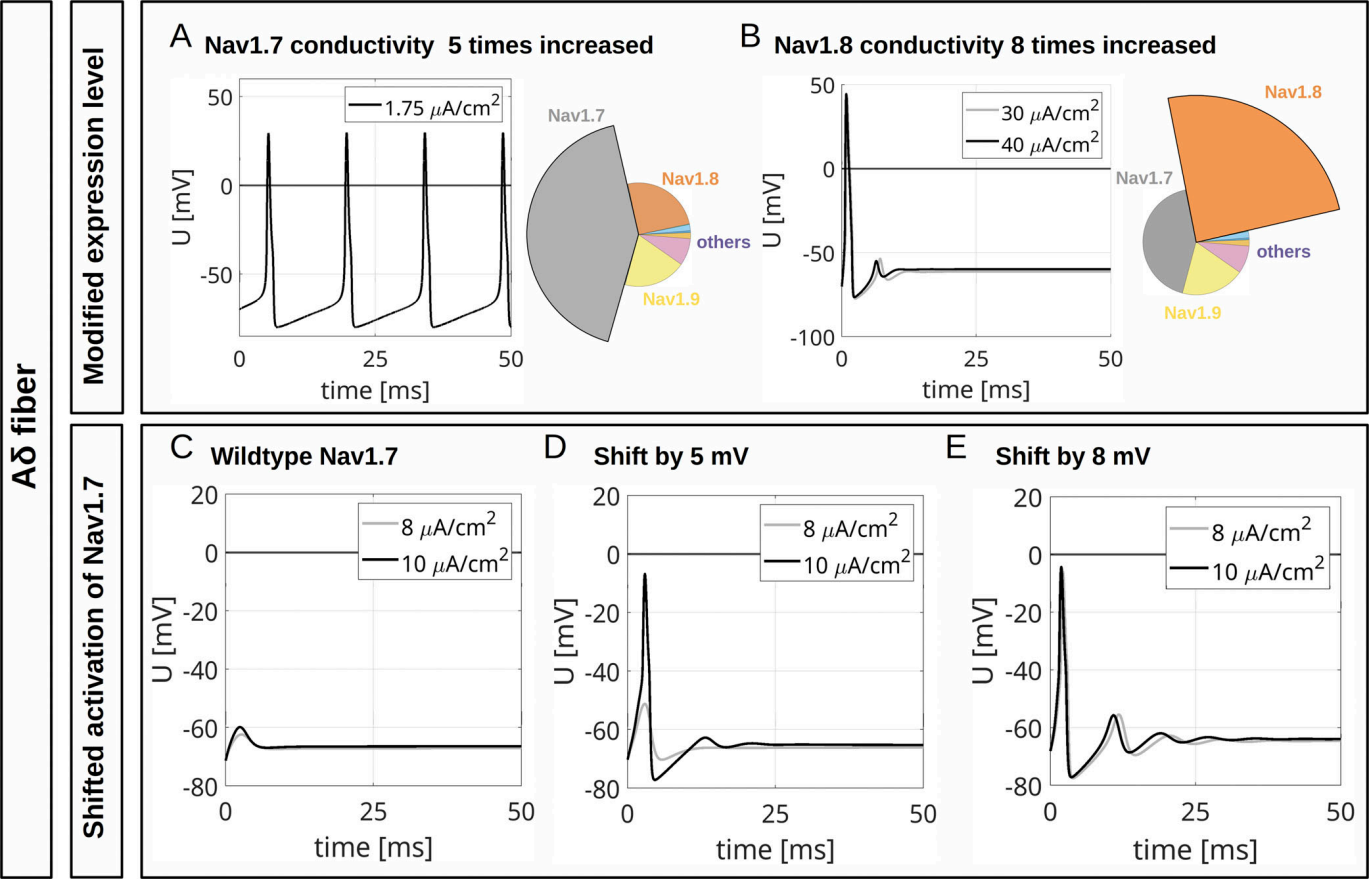
**Computational disease modelling in an Aδ fiber. (A)** Repetitive depolarizations resulting from increasing the maximal conductance for Na_v_1.7 to 500% of the WT value. The current injection of 1.75 μA/cm^2^ persists until the end of the simulation. **(B)** An increase of the maximal conductance for Na_v_1.8 to 800% of the WT value results in an isolated AP. The total *G*_*Na*_ in A and B are approximately the same. The current injections of 30 and 40 μA/cm^2^ persist until the end of the simulations. The pie charts indicate the abundance of different VGSC isoforms. **(C)** Current injections of 8 and 10 μA/cm^2^ trigger no APs in the simulated WT CMi (isoform abundances as in Fig. 10 A, upper left panel). **(D)** If the activation of Na_v_1.7 is shifted by 5 mV to hyperpolarized potentials, a current injection of 10 μA/cm^2^ but not of 8 μA/cm^2^ triggers an AP. **(E)** If the activation of Na_v_1.7 is shifted by 8 mV to hyperpolarized potentials, current injections of 8 and 10 μA/cm^2^ trigger an AP. The current injections in C–E persist until the end of the simulation.

When we increased Na_v_1.7 conduction by five times in our computer model, both the CMi model and the Aδ-fiber model showed persistent firing in response to an ongoing current injection of 1 and 1.75 µA/cm^2^, respectively (Fig. 11 A and Fig. 12 A). Interestingly, enhancing Na_v_1.8 conduction by 11 times in the CMi and by 8 times in the Aδ-fiber (revealing similar overall conductance as for a fivefold increased Na_v_1.7 conductance) was unable to induce ongoing AP firing (Fig. 11 B and Fig. 12 B). This finding supports the concept that the specific properties of Na_v_1.7 and not the increase of the total *G*_*Na*_ trigger the repetitive activity observed in Fig. 11 A and Fig. 12 A.

With unshifted Na_v_1.7 activation, subthreshold stimulations of 10 or 15 µA/cm^2^ did not evoke an AP in the CMi model (Fig. 11 C), and neither did subthreshold stimulations of 8 or 10 µA/cm^2^ in the Aδ-fiber model (Fig. 12 C). When Na_v_1.7 activation was shifted to hyperpolarized potentials by only 5 mV—which is often observed in erythromelalgia mutations (e.g., Lampert et al., 2009)—an AP was initiated by small stimulations, showing a reduced firing threshold (Fig. 11 D and Fig. 12 D). When shifted by 8 mV, weak stimulations even induced an AP followed by several dampened, repetitive depolarizations (Fig. 11 E and Fig. 12 E).

Taken together, our simulations, including Hodgkin–Huxley–like models of all nociceptive sodium channels, suggest a contribution of Na_v_1.9 to the fast upstroke of the AP and its shoulder formation and proved useful in modelling excitability changes associated with Na_v_1.7-linked chronic pain syndromes such as erythromelalgia.

## Discussion

In this study, we provide a detailed, comparative investigation of the gating properties of all VGSCs relevant in nociceptors with a focus on subthreshold depolarizations and propose computational models to predict their contributions to AP electrogenesis in two types of sensory neurons: CMi and Aδ-fibers. The models suggest that Na_v_1.9 may actively contribute to AP generation and formation of its shoulder in these neurons.

### VGSC isoforms are characterized by their distinct gating behavior in vitro and in silico

The TTXr channels measured in this study, i.e., Na_v_1.5 and Na_v_1.8, showed divergent gating properties with shifted voltage dependence of activation, SSFI, and ramp currents toward hyperpolarized and depolarized voltages, respectively. While Na_v_1.9 activation occurred at very hyperpolarized voltages, voltage dependence of Na_v_1.9 SSFI resembled the depolarized inactivation of Na_v_1.8. Out of the remaining TTXs channels, which all had their voltage dependence of activation, SSFI and ramp currents at subthreshold voltages, especially Na_v_1.3 and Na_v_1.7, elicited prominent currents in ramp and AP clamping. During the subthreshold phases of AP clamping, both displayed large AUC values and steeper current slopes compared with the other VGSCs tested. For Na_v_1.7, we observed slightly more hyperpolarized voltage dependences for both SSFI and ramp currents than for other TTXs channel isoforms, potentially enabling Na_v_1.7 to charge a cell at subthreshold or threshold voltages.

Our experiments emphasized the importance of Na_v_1.7 as an AP initiator, also since knocking out or enhancing Na_v_1.7 in silico led to omitted or multiple APs, respectively. This resembles putative loss*-* and gain-of-function pathophysiology that is attributed to Na_v_1.7 channelopathies (Dib-Hajj et al., 2005; Cox et al., 2006; Faber et al., 2012; Bennett et al., 2019; McDermott et al., 2019). Shifting activation of Na_v_1.7 to more hyperpolarized potentials in models for CMi or Aδ-fibers revealed the experimentally observed threshold reduction, suggesting the high potential of our simulations for disease modelling.

Our findings confirmed Na_v_1.8 contribution to the AP upstroke (Renganathan et al., 2001; Matsutomi et al., 2006; Rush et al., 2007; Vasylyev and Waxman, 2012), as it activates and fast-inactivates at more depolarized potentials, elicits large ramp currents at more depolarized potentials and shows little to no subthreshold activity in AP clamping compared with the other VGSCs tested. Interestingly, in our simulations including Na_v_1.9, the contribution of Na_v_1.8 to the shoulder was minor: knocking out Na_v_1.8 in the in silico experiments left APs in the CMi-model almost unchanged, while in the Aδ-model the overshoot was reduced. This is in line with findings from Na_v_1.8 knockout DRGs, where maximum AP voltages were lower compared with WT DRGs (Renganathan et al., 2001; Harty and Waxman, 2007). Yet, the initiation of the AP was not impeded when Na_v_1.8 was omitted from the established model.

The strongest difference between the two fibers modelled in this study is the inverse relative contribution of Na_v_1.7 compared with Na_v_1.8 during the AP: for the CMi model, Na_v_1.7 has the larger current and conductivity, whereas for the Aδ-fiber model it is Na_v_1.8. In an earlier modelling study, we investigated the contribution of Na_v_1.7, Na_v_1.8, and Na_v_1.9 to the excitability of a peripheral C-fiber to reproduce axonal spike propagation speed and activity-dependent slowing as recorded in microneurography experiments (Tigerholm et al., 2014; Tigerholm et al., 2015). We showed that the speed of two APs following each other depends on the influx of sodium through Na_v_1.7 relative to Na_v_1.8 current. Thus, fiber-specific biophysical properties may result from the specific contribution of the sodium channels expressed in their membrane.

Our simulations suggest that Na_v_1.9, in addition to its activation during rest, strongly contributes to the fast upstroke and is a major determinant of the shoulder of the AP. It represents by far the largest current and conductivity in our model of all integrated channels, and Nav1.9 currents were recorded in >80% of human DRG in a recent patch-clamp study (Zhang et al., 2024). Our findings are in line with current-clamp recordings on enteric neurons from Na_v_1.9 knockout mice, which show no shoulder in APs from mice lacking Na_v_1.9 (Osorio et al., 2014). An early report on the sensory neuron AP properties of those knockout mice revealed no differences in their AP width (Priest et al., 2005). The latter study did not differentiate between sensory neuron subtypes, yet in the realm of recent transcriptomic advances, we know that Na_v_1.9 expression differs strongly between subgroups (Tavares-Ferreira et al., 2022). It is also known that not all sensory neuron subtypes display a shoulder in their AP (Körner and Lampert, 2022). Thus, pooling the groups may have hidden the effect of Na_v_1.9 on the AP shoulder in this study. As Na_v_1.9 channelopathies are linked to both gain- and loss-of-pain syndromes (Leipold et al., 2013; Huang et al., 2017), clarification of the fiber type-specific role of Na_v_1.9 during AP generation could help to understand their complex pathophysiology.

We documented large Na_v_1.3 currents during clamping of an AP with a steep subthreshold voltage slope (AP3), and—consistent with literature—during steeper voltage ramps (Cummins et al., 2001). This might play a role in neuropathic pain, as—though only marginally expressed in C- and Aδ-fibers—some studies suggest that Na_v_1.3 is upregulated in spinal cord injuries or peripheral nerve injuries in rodents and therefore increases excitability (Hains et al., 2003; Hains et al., 2004; Lampert et al., 2006). Other studies present more ambiguous or even contradictory results, e.g., Na_v_1.3 knockout mice did not show lowered pain thresholds after nerve injury than WT mice (Lindia et al., 2005; Nassar et al., 2006).

Na_v_1.6 stood out to some extent, as it showed higher maximum inward currents and steeper current slopes during the subthreshold phase of AP clamping than other TTXs VGSC isoforms. Furthermore, Na_v_1.6 current was larger in the Aδ-fiber than in the CMi during the course of the AP. Yet, omitting Na_v_1.1, Na_v_1.2, and Na_v_1.6 during the in silico testing had no noticeable effect on AP morphology, likely because of their minor share in the spatial transcriptomics-derived expression patterns used in our model (Tavares-Ferreira et al., 2022). Na_v_1.6 has been linked to resurgent currents (O’Brien and Meisler, 2013), and these currents seem to be enhanced in A-fibers compared with C-fibers (Klinger et al., 2012). This is suggested as a reason why sea anemone toxin ATX-II led to painful sensations by enhancing resurgent currents in A-fibers, but not in C-fibers (Klinger et al., 2012).

Na_v_1.5 played no major role during subthreshold AP clamping and in contributing to the simulated AP, while it is also only marginally detected in C- and Aδ-fibers (Tavares-Ferreira et al., 2022). Due to its hyperpolarized activation and SSFI, it may be responding in cells with more hyperpolarized membrane potential. During development, Na_v_1.5 is the third TTXr conductance present and may have a more prominent role than observed in the two fiber types described here (reflecting the transcriptomic expression pattern of adult DRG donors). The role of Na_v_1.5 during development, injury, and regeneration needs to be investigated in more detail in follow-up studies (Renganathan et al., 2002; Tseng et al., 2010; Sousounis et al., 2020).

### Correlation between ramp current and window current is channel subtype specific

Slowly depolarizing voltage ramps induce ramp currents, mimicking the subthreshold phase of an AP. Ramp currents of WT channels have been examined for all VGSC isoforms individually (Cummins et al., 1998; Abriel et al., 2001; Herzog et al., 2003; El-Bizri et al., 2011; Power et al., 2012; Estacion and Waxman, 2013; DeCaen et al., 2014; Han et al., 2015a, 2015b; Zhang et al., 2017), but not yet in direct comparison, establishing experimenter-to-experimenter or lab-to-lab variations. Hereby, we provide an extensive and highly comparable data set of VGSC ramp currents.

While maximum ramp current size and AUC are predominantly influenced by the steepness of the inducing voltage ramp stimulus, VGSC subtypes rather influence the voltage dependence of ramp current responses and hence co-determine in which AP phase they contribute to its formation.

All tested TTXs channel isoforms elicited their peak ramp current at subthreshold voltages, enabling them to contribute to cell discharge prior to the AP threshold being exceeded. Of these subtypes, Na_v_1.3, Na_v_1.6, and Na_v_1.7 elicited the highest maximum ramp currents, corroborating their role as key players in subthreshold depolarization.

In VGSC literature, ramp and window currents were often assumed to describe a similar gating mechanism and were sometimes even used interchangeably (Wang et al., 1996; Magistretti and Alonso, 1999; Abriel et al., 2001; Blair and Bean, 2002; Enomoto et al., 2006; Matsutomi et al., 2006; Vasylyev and Waxman, 2012; Estacion and Waxman, 2013; Osteen et al., 2017). We here tested this hypothesis and showed that Na_v_1.1 and Na_v_1.2 (partly also Na_v_1.5, Na_v_1.7, and Na_v_1.8) showed a positive correlation between ramp current and window currents, suggesting a common underlying mechanism. Yet, because for Na_v_1.3 and Na_v_1.6 correlation was mostly nonexistent, we suppose that additional factors such as persistent current play into ramp current formation, as suggested previously (Estacion and Waxman, 2013).

### Expression systems affect in vitro VGSC measurements

Heterologous expression systems bear their own interpretation pitfalls, as they do not resemble the complex environment of primary neurons, e.g., regarding cell morphology, intercellular contacts, or membrane protein interactions. VGSC isoforms were shown to have different gating properties when expressed in different expression systems (Cummins et al., 2001; Rush et al., 2006a; Tan and Soderlund, 2009; Zhang et al., 2017).

In this study, we used both HEK and ND7/23 cells for electrophysiological VGSC examination. Multiple differences in their respective cell characteristics lead to subtype gating differences among each other having to be analyzed carefully. While endogenous Na_v_1.6 and Na_v_1.7 currents in ND7/23 can easily be suppressed by adding TTX to the ECS while measuring TTXr isoforms (Rogers et al., 2016; Lee et al., 2019), altered VGSC gating properties by endogenous expression of β1 and β3 subunits in ND7/23 cells cannot be eliminated (John et al., 2004; Lee et al., 2019). However, HEK cells themselves express β1A subunits endogenously (Moran et al., 2000). Therefore, comparisons between VGSC α subunit measurements have to be treated carefully, nonetheless and additional examination of α subunits in the presence of varying β subunits is encouraged. Furthermore, other cellular proteins such as fibroblast growth factor homologous factors, microtubule-associated proteins, or aquaporins have been shown to modify VGSC gating and/or current density (Wittmack et al., 2004; Rush et al., 2006b; Zhang and Verkman, 2010; O’Brien et al., 2012; Vanoye et al., 2013). Recent comparative studies of Na_v_1.2 and Na_v_1.6 variants also highlight the influence of alternative splicing on channel gating (Thompson et al., 2020; Vanoye et al., 2024).

Na_v_1.8 expression in HEK is challenging, and resulting currents are low (Gladwell et al., 1998; John et al., 2004). As in our hands, the expression of Na_v_1.8 in HEK293T cells yielded little to no current; we used ND7/23 cells, equivalent to the Na_v_1.9 measurements integrated into this study (Leipold et al., 2013; Leipold et al., 2015). Biophysical characteristics of Na_v_1.8 currents were shown to be similar between when expressed in ND7/23 cells or recorded from native DRG TTXr currents (John et al., 2004).

Even non-native neuronal cell lines such as ND7/23 do not resemble a comprehensive primary neuronal environment (Yin et al., 2016). Studies in expression systems closer to human peripheral sensory neurons, such as rodent or human DRG neurons, could give an even better insight into the channels of biophysics in vivo. Moreover, nociceptors derived from human iPSCs offer the possibility to investigate ion channels in an adaption of their native environment (Meents et al., 2019; Mis et al., 2019; Namer et al., 2019). This could also make up for the fact that we included neither co-expression of β-subunits into our measurements nor did we check for channel dimerization, both of which have been shown to alter biophysical properties of VGSCs (Brackenbury and Isom, 2011; Chahine and O’Leary, 2011; Clatot et al., 2017; Rühlmann et al., 2020).

Albeit our observations allow unprecedented comparison between isoforms as the experiments were conducted under the so-far best possible comparable conditions, it may be that differing expression systems do not model certain isoform specificities.

Biophysical differences, such as larger persistent or ramp currents, can occur between VGSC isoforms from different mammalian species (Browne et al., 2009; Tan and Soderlund, 2009; Han et al., 2015a). For Na_v_1.3 and Na_v_1.6 measurements in this study, we used rodent channel isoforms. Hence, complementary measurements with human channel isoforms are desirable.

In this study, as well as the studies providing the implemented Na_v_1.9 data, ICS contained CsF, which is widely used in patch-clamp experiments to enhance high-seal resistances for stable and long-lasting patch-clamp configurations (Kostyuk et al., 1975; Fernandez et al., 1984). Yet, usage of CsF has been shown to affect the gating of VGSCs in a subtype-specific manner. For example, the voltage dependence of activation and inactivation of Na_v_1.9 is shifted by about 15–20 mV to more hyperpolarized potentials in the presence of CsF (Rugiero et al., 2003; Coste et al., 2004). The gating of Na_v_1.3 and Na_v_1.7 is also affected by intracellular fluoride, albeit to a lesser extent (Chen et al., 2000; Meadows et al., 2002; Jarecki et al., 2008). While this does not necessarily limit subtype comparability within our in vitro measurements, which all included CsF, comparison to CsF-free experiments, such as measurements in primary neurons, has to be performed cautiously. Even minor shifts in channel biophysics can profoundly impact complex and therefore vulnerable computational models. Although the model established in this study provides valuable insights into the AP contribution of each channel, reissuing the model with data from CsF-free measurements would be desirable to increase its predictive power.

### Limitations of in silico modelling

Computational models are simplifications of the real world and therefore must be handled cautiously, especially when model complexity suggests the opposite. We estimated the distribution of the maximal *G*_*Na*_ per VGSC subunit by using mRNA expression data from a human DRG spatial transcriptomics study (Tavares-Ferreira et al., 2022). While this approach is closest to a quantitative measure currently accessible from human DRGs, mRNA expression levels do not always indicate that the respective channel is also translated to protein, trafficked to the cell surface, and fully functional. Even though correlation between mRNA expression, presence of VGSCs in the cell membrane, and biophysical properties has been suggested (Thériault and Chahine, 2014), alternative splicing, correct folding, and association with additional proteins or membrane turnover are just some factors that may impact channel translation from RNA to functional protein in the membrane (Buccitelli and Selbach, 2020). It is possible that mRNA levels do not directly relate to the amount of protein produced in the cells (Lindhout et al., 2020) and that we are overestimating or underestimating the conductance of the VGSCs modelled in this study. As proteomics of human DRGs, especially for VGSCs, is not yet available as a basis for our modelling, we used the published transcriptomics data to estimate the conductivity of the VGSC subtypes integrated into our simulations.

A promising solution to these problems is single-cell transcriptomics combined with functional analysis, such as Patch-seq, which offer the ability to link expression data directly to cell physiology (Lipovsek et al., 2021; Körner et al., 2022, 2024, *Preprint*) or single-cell proteomics, which are also currently developed for human nociceptors. Furthermore, since expression data are gathered at the cell soma, only limited assumptions can be made for small nerve fibers, e.g., in the human skin.

Several studies suggested the possibility of neuronal channel composition changing in compensation for loss- or gain-of-function mutations in VGSC isoforms, e.g., TTXs currents being upregulated in DRG neurons of Na_v_1.8 knockout mice (Akopian et al., 1999; Renganathan et al., 2000). Compensatory changes could also involve the expression of other ion channel types, e.g., calcium channels, or pathways indirectly affecting excitability. When modelling the effect of VGSC loss- or gain-of-function mutations in silico, these compensatory changes should be considered as soon as their extent is determined in precedent studies.

VGSC data are usually gathered at room temperature, as it was done in this study. However, the temperature dependence of VGSCs varies for each channel isoform and recorded voltages (Kriegeskorte et al., 2023). Thus, most computational models published in the recent years, including the one established in this study, do not accurately account for temperature, since this would require additional experimental data and re-parametrization of the models (Almog and Korngreen, 2016).

For fitting the gating variables of each channel subtype, we used current traces recorded at several potentials. At voltages below −40 mV, it often occurred that multiple parameter combinations described the currents successfully, creating some uncertainty (unidentifiability). Particularly in the case of Na_v_1.9, this may impact on simulation results.

Our computational model describes ion channels based on modified Hodgkin–Huxley dynamics and neglects spatial aspects. To further study the VGSC influence on AP generation, the VGSC isoforms should be implemented in a morphologically detailed C-fiber or Aδ-fiber model (Tigerholm et al., 2014; Tigerholm et al., 2019).

### VGSC isoform characterization can be the basis for assessing potential drug targets

In this study, we provide a broadly based groundwork for future comparative studies of VGSC isoforms, their biophysics, and interaction with each other. By feeding our measurements into a computational model, we generated an in silico tool to test hypotheses, investigate the contribution of each nociceptive sodium channel to AP genesis, and model pharmacology and the effect of disease-related mutations. This may also prove useful when developing new potential analgesics, such as specific channel blockers, or optimizing therapy schemes for chronic pain.

## Supplementary Material

Table S1 shows overview of all used cell lines and their culture media and supplements.

Table S2 shows duration of command voltage ramps.

Table S3 shows multiple comparisons of V50 values of voltage dependency of activation and fast inactivation measurement.

Table S4 shows multiple comparisons of slope values (k) of voltage dependency of activation and fast inactivation measurement.

Table S5 shows multiple comparisons of window current AUC and intersection voltage of activation and SSFI Boltzmann fit curve.

Table S6 shows multiple comparisons of fraction of channels remaining open after SSFI.

Table S7 shows descriptive statistics of parameters gathered from ramp current measurements.

Table S8 shows multiple comparisons of normalized maximum inward current values from ramp current measurements.

Table S9 shows multiple comparisons of the voltage dependence of maximum inward current from ramp current measurements.

Table S10 shows multiple comparisons of normalized AUC before maximum inward current values from ramp current measurements.

Table S11 shows multiple comparisons of normalized AUC before maximum inward current values from ramp current measurements.

Table S12 shows correlation of window current values and AUC values from ramp current measurements.

Table S13 shows descriptive statistics of parameters gathered from AP clamping measurements.

Table S14 shows multiple comparisons of parameters calculated from AP clamping measurements.

Appendix S1 shows in silico models.

## Data Availability

The code used for computational modelling is available at https://github.com/tstiehl/sodium-channels with commentary. All further data gathered in this study (including manual patch-clamp measurements and their analysis procedures) are available from the corresponding authors upon request.

## References

[bib1] Abriel, H., C. Cabo, X.H. Wehrens, I. Rivolta, H.K. Motoike, M. Memmi, C. Napolitano, S.G. Priori, and R.S. Kass. 2001. Novel arrhythmogenic mechanism revealed by a long-QT syndrome mutation in the cardiac Na(+) channel. Circ. Res. 88:740–745. 10.1161/hh0701.089668 11304498

[bib2] Ahern, C.A., J. Payandeh, F. Bosmans, and B. Chanda. 2016. The hitchhiker’s guide to the voltage-gated sodium channel galaxy. J. Gen. Physiol. 147:1–24. 10.1085/jgp.201511492 26712848 PMC4692491

[bib3] Akopian, A.N., V. Souslova, S. England, K. Okuse, N. Ogata, J. Ure, A. Smith, B.J. Kerr, S.B. McMahon, S. Boyce, . 1999. The tetrodotoxin-resistant sodium channel SNS has a specialized function in pain pathways. Nat. Neurosci. 2:541–548. 10.1038/9195 10448219

[bib4] Almog, M., and A. Korngreen. 2016. Is realistic neuronal modeling realistic? J. Neurophysiol. 116:2180–2209. 10.1152/jn.00360.2016 27535372 PMC5102320

[bib5] Bennett, D.L., A.J. Clark, J. Huang, S.G. Waxman, and S.D. Dib-Hajj. 2019. The role of voltage-gated sodium channels in pain signaling. Physiol. Rev. 99:1079–1151. 10.1152/physrev.00052.2017 30672368

[bib6] Bennett, D.L., and C.G. Woods. 2014. Painful and painless channelopathies. Lancet Neurol. 13:587–599. 10.1016/s1474-4422(14)70024-9 24813307

[bib7] Bhattacharjee, A., Y. Xiao, Z. Pei, and T.R. Cummins. 2018. Specialized sodium channels in pain transmission. *In* The Oxford Handbook of Neuronal Ion Channels. Oxford University Press, Oxford, UK. 10.1093/oxfordhb/9780190669164.013.3

[bib8] Bhuiyan, S.A., M. Xu, L. Yang, E. Semizoglou, P. Bhatia, K.I. Pantaleo, I. Tochitsky, A. Jain, B. Erdogan, S. Blair, . 2024. Harmonized cross-species cell atlases of trigeminal and dorsal root ganglia. Sci. Adv. 10:eadj9173. 10.1126/sciadv.adj9173 38905344 PMC11804847

[bib9] Blair, N.T., and B.P. Bean. 2002. Roles of tetrodotoxin (TTX)-sensitive Na^+^ current, TTX-resistant Na^+^ current, and Ca^2+^ current in the action potentials of nociceptive sensory neurons. J. Neurosci. 22:10277–10290. 10.1523/JNEUROSCI.22-23-10277.2002 12451128 PMC6758735

[bib10] Bothe, S.N., and A. Lampert. 2021. The insecticide deltamethrin enhances sodium channel slow inactivation of human Na_v_1.9, Na_v_1.8 and Na_v_1.7. Toxicol. Appl. Pharmacol. 428:115676. 10.1016/j.taap.2021.115676 34389319

[bib11] Brackenbury, W.J., and L.L. Isom. 2011. Na channel β subunits: Overachievers of the ion channel family. Front. Pharmacol. 2:53. 10.3389/fphar.2011.00053 22007171 PMC3181431

[bib12] Brouwer, B.A., I.S. Merkies, M.M. Gerrits, S.G. Waxman, J.G. Hoeijmakers, and C.G. Faber. 2014. Painful neuropathies: The emerging role of sodium channelopathies. J. Peripher. Nerv. Syst. 19:53–65. 10.1111/jns5.12071 25250524

[bib13] Browne, L.E., J.J. Clare, and D. Wray. 2009. Functional and pharmacological properties of human and rat NaV1.8 channels. Neuropharmacology. 56:905–914. 10.1016/j.neuropharm.2009.01.018 19371587

[bib14] Buccitelli, C., and M. Selbach. 2020. mRNAs, proteins and the emerging principles of gene expression control. Nat. Rev. Genet. 21:630–644. 10.1038/s41576-020-0258-4 32709985

[bib15] Catterall, W.A. 2023. Voltage gated sodium and calcium channels: Discovery, structure, function, and pharmacology. Channels. 17:2281714. 10.1080/19336950.2023.2281714 37983307 PMC10761118

[bib16] Catterall, W.A., A.L. Goldin, and S.G. Waxman. 2005. International Union of Pharmacology. XLVII. Nomenclature and structure-function relationships of voltage-gated sodium channels. Pharmacol. Rev. 57:397–409. 10.1124/pr.57.4.4 16382098

[bib17] Chahine, M., and M.E. O’Leary. 2011. Regulatory role of voltage-gated Na channel β subunits in sensory neurons. Front. Pharmacol. 2:70. 10.3389/fphar.2011.00070 22125538 PMC3221288

[bib18] Chen, Y.H., T.J. Dale, M.A. Romanos, W.R. Whitaker, X.M. Xie, and J.J. Clare. 2000. Cloning, distribution and functional analysis of the type III sodium channel from human brain. Eur. J. Neurosci. 12:4281–4289. 10.1111/j.1460-9568.2000.01336.x 11122339

[bib19] Clatot, J., M. Hoshi, X. Wan, H. Liu, A. Jain, K. Shinlapawittayatorn, C. Marionneau, E. Ficker, T. Ha, and I. Deschênes. 2017. Voltage-gated sodium channels assemble and gate as dimers. Nat. Commun. 8:2077. 10.1038/s41467-017-02262-0 29233994 PMC5727259

[bib20] Coste, B., N. Osorio, F. Padilla, M. Crest, and P. Delmas. 2004. Gating and modulation of presumptive NaV1.9 channels in enteric and spinal sensory neurons. Mol. Cell. Neurosci. 26:123–134. 10.1016/j.mcn.2004.01.015 15121184

[bib21] Cox, J.J., F. Reimann, A.K. Nicholas, G. Thornton, E. Roberts, K. Springell, G. Karbani, H. Jafri, J. Mannan, Y. Raashid, . 2006. An SCN9A channelopathy causes congenital inability to experience pain. Nature. 444:894–898. 10.1038/nature05413 17167479 PMC7212082

[bib22] Cummins, T.R., F. Aglieco, M. Renganathan, R.I. Herzog, S.D. Dib-Hajj, and S.G. Waxman. 2001. Nav1.3 sodium channels: Rapid repriming and slow closed-state inactivation display quantitative differences after expression in a mammalian cell line and in spinal sensory neurons. J. Neurosci. 21:5952–5961. 10.1523/JNEUROSCI.21-16-05952.2001 11487618 PMC6763143

[bib23] Cummins, T.R., J.R. Howe, and S.G. Waxman. 1998. Slow closed-state inactivation: A novel mechanism underlying ramp currents in cells expressing the hNE/PN1 sodium channel. J. Neurosci. 18:9607–9619. 10.1523/JNEUROSCI.18-23-09607.1998 9822722 PMC6793269

[bib24] de Lera Ruiz, M., and R.L. Kraus. 2015. Voltage-gated sodium channels: Structure, function, pharmacology, and clinical indications. J. Med. Chem. 58:7093–7118. 10.1021/jm501981g 25927480

[bib25] DeCaen, P.G., Y. Takahashi, T.A. Krulwich, M. Ito, and D.E. Clapham. 2014. Ionic selectivity and thermal adaptations within the voltage-gated sodium channel family of alkaliphilic Bacillus. Elife. 3:e04387. 10.7554/eLife.04387 25385530 PMC4225499

[bib26] Dib-Hajj, S.D., T.R. Cummins, J.A. Black, and S.G. Waxman. 2010. Sodium channels in normal and pathological pain. Annu. Rev. Neurosci. 33:325–347. 10.1146/annurev-neuro-060909-153234 20367448

[bib27] Dib-Hajj, S.D., P. Geha, and S.G. Waxman. 2017. Sodium channels in pain disorders: Pathophysiology and prospects for treatment. Pain. 158:S97–S107. 10.1097/j.pain.0000000000000854 28240647 PMC5350027

[bib28] Dib-Hajj, S.D., A.M. Rush, T.R. Cummins, F.M. Hisama, S. Novella, L. Tyrrell, L. Marshall, and S.G. Waxman. 2005. Gain-of-function mutation in Nav1.7 in familial erythromelalgia induces bursting of sensory neurons. Brain. 128:1847–1854. 10.1093/brain/awh514 15958509

[bib29] Eberhardt, E., S. Havlicek, D. Schmidt, A.S. Link, C. Neacsu, Z. Kohl, M. Hampl, A.M. Kist, A. Klinger, C. Nau, . 2015. Pattern of functional TTX-resistant sodium channels reveals a developmental stage of human iPSC- and ESC-derived nociceptors. Stem Cell Rep. 5:305–313. 10.1016/j.stemcr.2015.07.010 PMC4618592 26321143

[bib30] El-Bizri, N., K.M. Kahlig, J.C. Shyrock, A.L. George Jr., L. Belardinelli, and S. Rajamani. 2011. Ranolazine block of human Na v 1.4 sodium channels and paramyotonia congenita mutants. Channels. 5:161–172. 10.4161/chan.5.2.14851 21317558

[bib31] Enomoto, A., J.M. Han, C.-F. Hsiao, N. Wu, and S.H. Chandler. 2006. Participation of sodium currents in burst generation and control of membrane excitability in mesencephalic trigeminal neurons. J. Neurosci. 26:3412–3422. 10.1523/JNEUROSCI.5274-05.2006 16571748 PMC6673852

[bib32] Estacion, M., and S.G. Waxman. 2013. The response of Na(V)1.3 sodium channels to ramp stimuli: Multiple components and mechanisms. J. Neurophysiol. 109:306–314. 10.1152/jn.00438.2012 23114218

[bib33] Faber, C.G., J.G. Hoeijmakers, H.S. Ahn, X. Cheng, C. Han, J.S. Choi, M. Estacion, G. Lauria, E.K. Vanhoutte, M.M. Gerrits, . 2012. Gain of function Na_v_1.7 mutations in idiopathic small fiber neuropathy. Ann. Neurol. 71:26–39. 10.1002/ana.22485 21698661

[bib34] Felts, P.A., S. Yokoyama, S. Dib-Hajj, J.A. Black, and S.G. Waxman. 1997. Sodium channel alpha-subunit mRNAs I, II, III, NaG, Na6 and hNE (PN1): Different expression patterns in developing rat nervous system. Brain Res. Mol. Brain Res. 45:71–82. 10.1016/s0169-328x(96)00241-0 9105672

[bib35] Fernandez, J.M., A.P. Fox, and S. Krasne. 1984. Membrane patches and whole-cell membranes: A comparison of electrical properties in rat clonal pituitary (GH3) cells. J. Physiol. 356:565–585. 10.1113/jphysiol.1984.sp015483 6097678 PMC1193182

[bib36] Fertleman, C.R., M.D. Baker, K.A. Parker, S. Moffatt, F.V. Elmslie, B. Abrahamsen, J. Ostman, N. Klugbauer, J.N. Wood, R.M. Gardiner, and M. Rees. 2006. SCN9A mutations in paroxysmal extreme pain disorder: Allelic variants underlie distinct channel defects and phenotypes. Neuron. 52:767–774. 10.1016/j.neuron.2006.10.006 17145499

[bib37] Fertleman, C.R., C.D. Ferrie, J. Aicardi, N.A. Bednarek, O. Eeg-Olofsson, F.V. Elmslie, D.A. Griesemer, F. Goutières, M. Kirkpatrick, I.N. Malmros, . 2007. Paroxysmal extreme pain disorder (previously familial rectal pain syndrome). Neurology. 69:586–595. 10.1212/01.wnl.0000268065.16865.5f 17679678

[bib38] Fukuoka, T., K. Kobayashi, H. Yamanaka, K. Obata, Y. Dai, and K. Noguchi. 2008. Comparative study of the distribution of the alpha-subunits of voltage-gated sodium channels in normal and axotomized rat dorsal root ganglion neurons. J. Comp. Neurol. 510:188–206. 10.1002/cne.21786 18615542

[bib39] Gladwell, Z., D. Trezise, C. Plumpton, X. Xie, and S. Tate. 1998. Expression and functional analysis of recombinant rat sensory neuron specific Na^+^ channels in a mammalian cell line. J. Physiol. 513:141P.

[bib40] Goodwin, G., and S.B. McMahon. 2021. The physiological function of different voltage-gated sodium channels in pain. Nat. Rev. Neurosci. 22:263–274. 10.1038/s41583-021-00444-w 33782571

[bib41] Goral, R.O., E. Leipold, E. Nematian-Ardestani, and S.H. Heinemann. 2015. Heterologous expression of NaV1.9 chimeras in various cell systems. Pflugers Arch. 467:2423–2435. 10.1007/s00424-015-1709-1 25916202

[bib42] Hains, B.C., J.P. Klein, C.Y. Saab, M.J. Craner, J.A. Black, and S.G. Waxman. 2003. Upregulation of sodium channel Na_v_1.3 and functional involvement in neuronal hyperexcitability associated with central neuropathic pain after spinal cord injury. J. Neurosci. 23:8881–8892. 10.1523/jneurosci.23-26-08881.2003 14523090 PMC6740400

[bib43] Hains, B.C., C.Y. Saab, J.P. Klein, M.J. Craner, and S.G. Waxman. 2004. Altered sodium channel expression in second-order spinal sensory neurons contributes to pain after peripheral nerve injury. J. Neurosci. 24:4832–4839. 10.1523/jneurosci.0300-04.2004 15152043 PMC6729453

[bib44] Han, C., M. Estacion, J. Huang, D. Vasylyev, P. Zhao, S.D. Dib-Hajj, and S.G. Waxman. 2015a. Human Na(v)1.8: Enhanced persistent and ramp currents contribute to distinct firing properties of human DRG neurons. J. Neurophysiol. 113:3172–3185. 10.1152/jn.00113.2015 25787950 PMC4432682

[bib45] Han, C., Y. Yang, B.T.A. de Greef, J.G.J. Hoeijmakers, M.M. Gerrits, C. Verhamme, J. Qu, G. Lauria, I.S.J. Merkies, C.G. Faber, . 2015b. The domain II S4-S5 linker in Nav1.9: A missense mutation enhances activation, impairs fast inactivation, and produces human painful neuropathy. Neuromol. Med. 17:158–169. 10.1007/s12017-015-8347-9 25791876

[bib46] Harty, T.P., and S.G. Waxman. 2007. Inactivation properties of Sodium Channel Nav1.8 maintain action potential amplitude in small DRG neurons in the context of depolarization. Mol. Pain. 3:12. 10.1186/1744-8069-3-12 17540018 PMC1892009

[bib47] Herzog, R.I., T.R. Cummins, F. Ghassemi, S.D. Dib-Hajj, and S.G. Waxman. 2003. Distinct repriming and closed-state inactivation kinetics of Nav1.6 and Nav1.7 sodium channels in mouse spinal sensory neurons. J. Physiol. 551:741–750. 10.1113/jphysiol.2003.047357 12843211 PMC2343279

[bib48] Herzog, R.I., T.R. Cummins, and S.G. Waxman. 2001. Persistent TTX-resistant Na^+^ current affects resting potential and response to depolarization in simulated spinal sensory neurons. J. Neurophysiol. 86:1351–1364. 10.1152/jn.2001.86.3.1351 11535682

[bib49] Hodgkin, A.L., and A.F. Huxley. 1952. A quantitative description of membrane current and its application to conduction and excitation in nerve. J. Physiol. 117:500–544. 10.1113/jphysiol.1952.sp004764 12991237 PMC1392413

[bib50] Huang, J., C.G. Vanoye, A. Cutts, Y.P. Goldberg, S.D. Dib-Hajj, C.J. Cohen, S.G. Waxman, and A.L. George Jr. 2017. Sodium channel NaV1.9 mutations associated with insensitivity to pain dampen neuronal excitability. J. Clin. Invest. 127:2805–2814. 10.1172/JCI92373 28530638 PMC5490760

[bib51] Jarecki, B.W., P.L. Sheets, J.O. Jackson II, and T.R. Cummins. 2008. Paroxysmal extreme pain disorder mutations within the D3/S4-S5 linker of Nav1.7 cause moderate destabilization of fast inactivation. J. Physiol. 586:4137–4153. 10.1113/jphysiol.2008.154906 18599537 PMC2652197

[bib52] John, V.H., M.J. Main, A.J. Powell, Z.M. Gladwell, C. Hick, H.S. Sidhu, J.J. Clare, S. Tate, and D.J. Trezise. 2004. Heterologous expression and functional analysis of rat Nav1.8 (SNS) voltage-gated sodium channels in the dorsal root ganglion neuroblastoma cell line ND7-23. Neuropharmacology. 46:425–438. 10.1016/j.neuropharm.2003.09.018 14975698

[bib53] Jung, M., M. Dourado, J. Maksymetz, A. Jacobson, B.I. Laufer, M. Baca, O. Foreman, D.H. Hackos, L. Riol-Blanco, and J.S. Kaminker. 2023. Cross-species transcriptomic atlas of dorsal root ganglia reveals species-specific programs for sensory function. Nat. Commun. 14:366. 10.1038/s41467-023-36014-0 36690629 PMC9870891

[bib54] Kleggetveit, I.P., B. Namer, R. Schmidt, T. Helås, M. Rückel, K. Ørstavik, M. Schmelz, and E. Jørum. 2012. High spontaneous activity of C-nociceptors in painful polyneuropathy. Pain. 153:2040–2047. 10.1016/j.pain.2012.05.017 22986070

[bib55] Klinger, A.B., M. Eberhardt, A.S. Link, B. Namer, L.K. Kutsche, E.T. Schuy, R. Sittl, T. Hoffmann, C. Alzheimer, T. Huth, . 2012. Sea-anemone toxin ATX-II elicits A-fiber-dependent pain and enhances resurgent and persistent sodium currents in large sensory neurons. Mol. Pain. 8:69. 10.1186/1744-8069-8-69 22978421 PMC3495684

[bib56] Klugbauer, N., L. Lacinova, V. Flockerzi, and F. Hofmann. 1995. Structure and functional expression of a new member of the tetrodotoxin-sensitive voltage-activated sodium channel family from human neuroendocrine cells. EMBO J. 14:1084–1090. 10.1002/j.1460-2075.1995.tb07091.x 7720699 PMC398185

[bib57] Körner, J., N. Haag, I. Kurth, and A. Lampert. 2022. Genetics meets function in sodium channel-related pain disorders. Neuroforum. 28:67–75. 10.1515/nf-2021-0035

[bib58] Körner, J., and A. Lampert. 2020. Sodium channels. *In* The Senses - A Comprehensive Reference. B. Fritzsch, editor. Academic Press, Cambridge, MA, USA. 120–141. 10.1016/B978-0-12-809324-5.24208-9

[bib59] Körner, J., and A. Lampert. 2022. Functional subgroups of rat and human sensory neurons: A systematic review of electrophysiological properties. Pflugers Arch. 474:367–385. 10.1007/s00424-021-02656-6 35031856 PMC8924089

[bib139] Körner, J., D. Howard, H.J. Solinski, M.M. Moreno, N. Haag, A. Fiebig, S.A. Bhuiyan, I. Tuklucu, R. Bott, I. Sankaranarayanan, . 2024. Molecular architecture of human dermal sleeping nociceptors. bioRxiv. 10.1101/2024.12.20.629638 (Preprint posted December 20, 2024)

[bib60] Körner, J., J. Meents, J.P. Machtens, and A. Lampert. 2018. β1 subunit stabilises sodium channel Nav1.7 against mechanical stress. J. Physiol. 596:2433–2445. 10.1113/JP275905 29659026 PMC6002208

[bib61] Kostyuk, P.G., O.A. Krishtal, and V.I. Pidoplichko. 1975. Effect of internal fluoride and phosphate on membrane currents during intracellular dialysis of nerve cells. Nature. 257:691–693. 10.1038/257691a0 1186845

[bib62] Kriegeskorte, S., R. Bott, M. Hampl, A. Korngreen, R. Hausmann, and A. Lampert. 2023. Cold and warmth intensify pain-linked sodium channel gating effects and persistent currents. J. Gen. Physiol. 155:e202213312. 10.1085/jgp.202213312 37531097 PMC10397059

[bib63] Kupari, J., D. Usoskin, M. Parisien, D. Lou, Y. Hu, M. Fatt, P. Lönnerberg, M. Spångberg, B. Eriksson, N. Barkas, . 2021. Single cell transcriptomics of primate sensory neurons identifies cell types associated with chronic pain. Nat. Commun. 12:1510. 10.1038/s41467-021-21725-z 33686078 PMC7940623

[bib64] Laezza, F., A. Lampert, M.A. Kozel, B.R. Gerber, A.M. Rush, J.M. Nerbonne, S.G. Waxman, S.D. Dib-Hajj, and D.M. Ornitz. 2009. FGF14 N-terminal splice variants differentially modulate Nav1.2 and Nav1.6-encoded sodium channels. Mol. Cell. Neurosci. 42:90–101. 10.1016/j.mcn.2009.05.007 19465131 PMC2832592

[bib65] Lampert, A., S.D. Dib-Hajj, E.M. Eastman, L. Tyrrell, Z. Lin, Y. Yang, and S.G. Waxman. 2009. Erythromelalgia mutation L823R shifts activation and inactivation of threshold sodium channel Nav1.7 to hyperpolarized potentials. Biochem. Biophys. Res. Commun. 390:319–324. 10.1016/j.bbrc.2009.09.121 19800314

[bib66] Lampert, A., M. Eberhardt, and S.G. Waxman. 2014. Altered sodium channel gating as molecular basis for pain: Contribution of activation, inactivation, and resurgent currents. Handb. Exp. Pharmacol. 221:91–110. 10.1007/978-3-642-41588-3_5 24737233

[bib67] Lampert, A., B.C. Hains, and S.G. Waxman. 2006. Upregulation of persistent and ramp sodium current in dorsal horn neurons after spinal cord injury. Exp. Brain Res. 174:660–666. 10.1007/s00221-006-0511-x 16718433

[bib68] Lampert, A., A.O. O’Reilly, P. Reeh, and A. Leffler. 2010. Sodium channelopathies and pain. Pflugers Arch. 460:249–263. 10.1007/s00424-009-0779-3 20101409

[bib69] Lee, J., S. Kim, H.M. Kim, H.J. Kim, and F.H. Yu. 2019. NaV1.6 and NaV1.7 channels are major endogenous voltage-gated sodium channels in ND7/23 cells. PLoS One. 14:e0221156. 10.1371/journal.pone.0221156 31419255 PMC6697327

[bib70] Leipold, E., A. Hanson-Kahn, M. Frick, P. Gong, J.A. Bernstein, M. Voigt, I. Katona, R. Oliver Goral, J. Altmüller, P. Nürnberg, . 2015. Cold-aggravated pain in humans caused by a hyperactive NaV1.9 channel mutant. Nat. Commun. 6:10049. 10.1038/ncomms10049 26645915 PMC4686659

[bib71] Leipold, E., L. Liebmann, G.C. Korenke, T. Heinrich, S. Giesselmann, J. Baets, M. Ebbinghaus, R.O. Goral, T. Stödberg, J.C. Hennings, . 2013. A de novo gain-of-function mutation in SCN11A causes loss of pain perception. Nat. Genet. 45:1399–1404. 10.1038/ng.2767 24036948

[bib72] Liang, L., S. Fazel Darbandi, S. Pochareddy, F.O. Gulden, M.C. Gilson, B.K. Sheppard, A. Sahagun, J.Y. An, D.M. Werling, J.L.R. Rubenstein, . 2021. Developmental dynamics of voltage-gated sodium channel isoform expression in the human and mouse brain. Genome Med. 13:135. 10.1186/s13073-021-00949-0 34425903 PMC8383430

[bib73] Lindhout, F.W., R. Kooistra, S. Portegies, L.J. Herstel, R. Stucchi, B.L. Snoek, A.M. Altelaar, H.D. MacGillavry, C.J. Wierenga, and C.C. Hoogenraad. 2020. Quantitative mapping of transcriptome and proteome dynamics during polarization of human iPSC-derived neurons. Elife. 9:e58124. 10.7554/eLife.58124 32940601 PMC7498259

[bib74] Lindia, J.A., M.G. Köhler, W.J. Martin, and C. Abbadie. 2005. Relationship between sodium channel NaV1.3 expression and neuropathic pain behavior in rats. Pain. 117:145–153. 10.1016/j.pain.2005.05.027 16061326

[bib75] Lipovsek, M., C. Bardy, C.R. Cadwell, K. Hadley, D. Kobak, and S.J. Tripathy. 2021. Patch-seq: Past, present, and future. J. Neurosci. 41:937–946. 10.1523/jneurosci.1653-20.2020 33431632 PMC7880286

[bib76] Magistretti, J., and A. Alonso. 1999. Biophysical properties and slow voltage-dependent inactivation of a sustained sodium current in entorhinal cortex layer-II principal neurons: A whole-cell and single-channel study. J. Gen. Physiol. 114:491–509. 10.1085/jgp.114.4.491 10498669 PMC2229464

[bib77] Maingret, F., B. Coste, F. Padilla, N. Clerc, M. Crest, S.M. Korogod, and P. Delmas. 2008. Inflammatory mediators increase Nav1.9 current and excitability in nociceptors through a coincident detection mechanism. J. Gen. Physiol. 131:211–225. 10.1085/jgp.200709935 18270172 PMC2248717

[bib78] Matsutomi, T., C. Nakamoto, T. Zheng, J. Kakimura, and N. Ogata. 2006. Multiple types of Na(+) currents mediate action potential electrogenesis in small neurons of mouse dorsal root ganglia. Pflugers Arch. 453:83–96. 10.1007/s00424-006-0104-3 16838161

[bib79] Maxion, A., E. Kutafina, M.F. Dohrn, P. Sacré, A. Lampert, J. Tigerholm, and B. Namer. 2023. A modelling study to dissect the potential role of voltage-gated ion channels in activity-dependent conduction velocity changes as identified in small fiber neuropathy patients. Front. Comput. Neurosci. 17:1265958. 10.3389/fncom.2023.1265958 38156040 PMC10752960

[bib80] McDermott, L.A., G.A. Weir, A.C. Themistocleous, A.R. Segerdahl, I. Blesneac, G. Baskozos, A.J. Clark, V. Millar, L.J. Peck, D. Ebner, . 2019. Defining the functional role of Na_V_1.7 in human nociception. Neuron. 101:905–919.e8. 10.1016/j.neuron.2019.01.047 30795902 PMC6424805

[bib81] Meadows, L.S., Y.H. Chen, A.J. Powell, J.J. Clare, and D.S. Ragsdale. 2002. Functional modulation of human brain Nav1.3 sodium channels, expressed in mammalian cells, by auxiliary beta 1, beta 2 and beta 3 subunits. Neuroscience. 114:745–753. 10.1016/s0306-4522(02)00242-7 12220575

[bib82] Meents, J.E., E. Bressan, S. Sontag, A. Foerster, P. Hautvast, C. Rösseler, M. Hampl, H. Schüler, R. Goetzke, T.K.C. Le, . 2019. The role of Nav1.7 in human nociceptors: Insights from human induced pluripotent stem cell-derived sensory neurons of erythromelalgia patients. Pain. 160:1327–1341. 10.1097/j.pain.0000000000001511 30720580 PMC6554007

[bib83] Mis, M.A., Y. Yang, B.S. Tanaka, C. Gomis-Perez, S. Liu, F. Dib-Hajj, T. Adi, R. Garcia-Milian, B.R. Schulman, S.D. Dib-Hajj, and S.G. Waxman. 2019. Resilience to pain: A peripheral component identified using induced pluripotent stem cells and dynamic clamp. J. Neurosci. 39:382–392. 10.1523/jneurosci.2433-18.2018 30459225 PMC6335750

[bib84] Moran, O., M. Nizzari, and F. Conti. 2000. Endogenous expression of the β1A sodium channel subunit in HEK-293 cells. FEBS Lett. 473:132–134. 10.1016/S0014-5793(00)01518-0 10812059

[bib85] Motulsky, H.J., and R.E. Brown. 2006. Detecting outliers when fitting data with nonlinear regression - a new method based on robust nonlinear regression and the false discovery rate. BMC Bioinformatics. 7:123. 10.1186/1471-2105-7-123 16526949 PMC1472692

[bib86] Namer, B., and H.O. Handwerker. 2009. Translational nociceptor research as guide to human pain perceptions and pathophysiology. Exp. Brain Res. 196:163–172. 10.1007/s00221-009-1777-6 19350229

[bib87] Namer, B., D. Schmidt, E. Eberhardt, M. Maroni, E. Dorfmeister, I.P. Kleggetveit, L. Kaluza, J. Meents, A. Gerlach, Z. Lin, . 2019. Pain relief in a neuropathy patient by lacosamide: Proof of principle of clinical translation from patient-specific iPS cell-derived nociceptors. EBioMedicine. 39:401–408. 10.1016/j.ebiom.2018.11.042 30503201 PMC6354557

[bib88] Nassar, M.A., M.D. Baker, A. Levato, R. Ingram, G. Mallucci, S.B. McMahon, and J.N. Wood. 2006. Nerve injury induces robust allodynia and ectopic discharges in Nav1.3 null mutant mice. Mol. Pain. 2:33. 10.1186/1744-8069-2-33 17052333 PMC1630424

[bib89] Nguyen, M.Q., L.J. von Buchholtz, A.N. Reker, N.J. Ryba, and S. Davidson. 2021. Single-nucleus transcriptomic analysis of human dorsal root ganglion neurons. Elife. 10:e71752. 10.7554/eLife.71752 34825887 PMC8626086

[bib90] O’Brien, J.E., and M.H. Meisler. 2013. Sodium channel SCN8A (Nav1.6): properties and de novo mutations in epileptic encephalopathy and intellectual disability. Front. Genet. 4:213. 10.3389/fgene.2013.00213 24194747 PMC3809569

[bib91] O’Brien, J.E., L.M. Sharkey, C.N. Vallianatos, C. Han, J.C. Blossom, T. Yu, S.G. Waxman, S.D. Dib-Hajj, and M.H. Meisler. 2012. Interaction of voltage-gated sodium channel Nav1.6 (SCN8A) with microtubule-associated protein Map1b. J. Biol. Chem. 287:18459–18466. 10.1074/jbc.M111.336024 22474336 PMC3365756

[bib92] Osorio, N., S. Korogod, and P. Delmas. 2014. Specialized functions of Nav1.5 and Nav1.9 channels in electrogenesis of myenteric neurons in intact mouse ganglia. J. Neurosci. 34:5233–5244. 10.1523/JNEUROSCI.0057-14.2014 24719102 PMC6609004

[bib93] Osteen, J.D., K. Sampson, V. Iyer, D. Julius, and F. Bosmans. 2017. Pharmacology of the Na_v_1.1 domain IV voltage sensor reveals coupling between inactivation gating processes. Proc. Natl. Acad. Sci. USA. 114:6836–6841. 10.1073/pnas.1621263114 28607094 PMC5495237

[bib94] Petersson, M.E., O. Obreja, A. Lampert, R.W. Carr, M. Schmelz, and E. Fransén. 2014. Differential axonal conduction patterns of mechano-sensitive and mechano-insensitive nociceptors--a combined experimental and modelling study. PLoS One. 9:e103556. 10.1371/journal.pone.0103556 25136824 PMC4138079

[bib95] Power, K.E., K.P. Carlin, and B. Fedirchuk. 2012. Modulation of voltage-gated sodium channels hyperpolarizes the voltage threshold for activation in spinal motoneurones. Exp. Brain Res. 217:311–322. 10.1007/s00221-011-2994-3 22218500

[bib96] Priest, B.T., B.A. Murphy, J.A. Lindia, C. Diaz, C. Abbadie, A.M. Ritter, P. Liberator, L.M. Iyer, S.F. Kash, M.G. Kohler, . 2005. Contribution of the tetrodotoxin-resistant voltage-gated sodium channel NaV1.9 to sensory transmission and nociceptive behavior. Proc. Natl. Acad. Sci. USA. 102:9382–9387. 10.1073/pnas.0501549102 15964986 PMC1166597

[bib97] Renganathan, M., T.R. Cummins, W.N. Hormuzdiar, and S.G. Waxman. 2000. alpha-SNS produces the slow TTX-resistant sodium current in large cutaneous afferent DRG neurons. J. Neurophysiol. 84:710–718. 10.1152/jn.2000.84.2.710 10938298

[bib98] Renganathan, M., T.R. Cummins, and S.G. Waxman. 2001. Contribution of Na(v)1.8 sodium channels to action potential electrogenesis in DRG neurons. J. Neurophysiol. 86:629–640. 10.1152/jn.2001.86.2.629 11495938

[bib99] Renganathan, M., S. Dib-Hajj, and S.G. Waxman. 2002. Na(v)1.5 underlies the “third TTX-R sodium current” in rat small DRG neurons. Brain Res. Mol. Brain Res. 106:70–82. 10.1016/S0169-328X(02)00411-4 12393266

[bib100] Rogers, M., N. Zidar, D. Kikelj, and R.W. Kirby. 2016. Characterization of endogenous sodium channels in the ND7-23 neuroblastoma cell line: Implications for use as a heterologous ion channel expression system suitable for automated patch clamp screening. Assay Drug Dev. Technol. 14:109–130. 10.1089/adt.2016.704 26991361 PMC4800267

[bib101] Rugiero, F., M. Mistry, D. Sage, J.A. Black, S.G. Waxman, M. Crest, N. Clerc, P. Delmas, and M. Gola. 2003. Selective expression of a persistent tetrodotoxin-resistant Na^+^ current and NaV1.9 subunit in myenteric sensory neurons. J. Neurosci. 23:2715–2725. 10.1523/jneurosci.23-07-02715.2003 12684457 PMC6742082

[bib102] Rühlmann, A.H., J. Körner, R. Hausmann, N. Bebrivenski, C. Neuhof, S. Detro-Dassen, P. Hautvast, C.A. Benasolo, J. Meents, J.P. Machtens, . 2020. Uncoupling sodium channel dimers restores the phenotype of a pain-linked Na_v_ 1.7 channel mutation. Br. J. Pharmacol. 177:4481–4496. 10.1111/bph.15196 32663327 PMC7484505

[bib103] Rush, A.M., T.R. Cummins, and S.G. Waxman. 2007. Multiple sodium channels and their roles in electrogenesis within dorsal root ganglion neurons. J. Physiol. 579:1–14. 10.1113/jphysiol.2006.121483 17158175 PMC2075388

[bib104] Rush, A.M., S.D. Dib-Hajj, S. Liu, T.R. Cummins, J.A. Black, and S.G. Waxman. 2006a. A single sodium channel mutation produces hyper- or hypoexcitability in different types of neurons. Proc. Natl. Acad. Sci. USA. 103:8245–8250. 10.1073/pnas.0602813103 16702558 PMC1472458

[bib105] Rush, A.M., E.K. Wittmack, L. Tyrrell, J.A. Black, S.D. Dib-Hajj, and S.G. Waxman. 2006b. Differential modulation of sodium channel Na(v)1.6 by two members of the fibroblast growth factor homologous factor 2 subfamily. Eur. J. Neurosci. 23:2551–2562. 10.1111/j.1460-9568.2006.04789.x 16817858

[bib106] Sheets, M.F., and D.A. Hanck. 1999. Gating of skeletal and cardiac muscle sodium channels in mammalian cells. J. Physiol. 514:425–436. 10.1111/j.1469-7793.1999.425ae.x 9852324 PMC2269069

[bib107] Sizova, D.V., J. Huang, E.J. Akin, M. Estacion, C. Gomis-Perez, S.G. Waxman, and S.D. Dib-Hajj. 2020. A 49-residue sequence motif in the C terminus of Nav1.9 regulates trafficking of the channel to the plasma membrane. J. Biol. Chem. 295:1077–1090. 10.1074/jbc.RA119.011424 31822564 PMC6983848

[bib108] Sousounis, K., B. Erdogan, M. Levin, and J.L. Whited. 2020. Precise control of ion channel and gap junction expression is required for patterning of the regenerating axolotl limb. Int. J. Dev. Biol. 64:485–494. 10.1387/ijdb.200114jw 33200809 PMC8796139

[bib109] Stępień, A., D. Sałacińska, J. Staszewski, M. Durka-Kęsy, and J. Dobrogowski. 2020. Paroxysmal extreme pain disorder in family with c.3892G > T (p.Val1298Phe) in the SCN9A gene mutation - case report. BMC Neurol. 20:182. 10.1186/s12883-020-01770-9 32404070 PMC7218613

[bib110] Stiles, P.J., and C.G. Gray. 2021. Improved Hodgkin-Huxley type model for neural action potentials. Eur. Biophys. J. 50:819–828. 10.1007/s00249-021-01547-z 34181052

[bib111] Tan, J., and D.M. Soderlund. 2009. Human and rat Nav1.3 voltage-gated sodium channels differ in inactivation properties and sensitivity to the pyrethroid insecticide tefluthrin. Neurotoxicology. 30:81–89. 10.1016/j.neuro.2008.10.008 19026681 PMC2696113

[bib112] Tang, Z., Z. Chen, B. Tang, and H. Jiang. 2015. Primary erythromelalgia: A review. Orphanet J. Rare Dis. 10:127. 10.1186/s13023-015-0347-1 26419464 PMC4589109

[bib113] Tate, S., S. Benn, C. Hick, D. Trezise, V. John, R.J. Mannion, M. Costigan, C. Plumpton, D. Grose, Z. Gladwell, . 1998. Two sodium channels contribute to the TTX-R sodium current in primary sensory neurons. Nat. Neurosci. 1:653–655. 10.1038/3652 10196578

[bib114] Tavares-Ferreira, D., S. Shiers, P.R. Ray, A. Wangzhou, V. Jeevakumar, I. Sankaranarayanan, A.M. Cervantes, J.C. Reese, A. Chamessian, B.A. Copits, . 2022. Spatial transcriptomics of dorsal root ganglia identifies molecular signatures of human nociceptors. Sci. Transl. Med. 14:eabj8186. 10.1126/scitranslmed.abj8186 35171654 PMC9272153

[bib115] Thériault, O., and M. Chahine. 2014. Correlation of the electrophysiological profiles and sodium channel transcripts of individual rat dorsal root ganglia neurons. Front. Cell. Neurosci. 8:285. 10.3389/fncel.2014.00285 25285069 PMC4168718

[bib116] Thompson, C.H., R. Ben-Shalom, K.J. Bender, and A.L. George Jr. 2020. Alternative splicing potentiates dysfunction of early-onset epileptic encephalopathy SCN2A variants. J. Gen. Physiol. 152:e201912442. 10.1085/jgp.201912442 31995133 PMC7054859

[bib117] Thompson, C.H., F. Potet, T.V. Abramova, J.-M. DeKeyser, N.F. Ghabra, C.G. Vanoye, J.J. Millichap, and A.L. George Jr. 2023. Epilepsy-associated SCN2A (NaV1.2) variants exhibit diverse and complex functional properties. J. Gen. Physiol. 155:e202313375. 10.1085/jgp.202313375 37578743 PMC10424433

[bib118] Tian, J., A.G. Bavencoffe, M.X. Zhu, and E.T. Walters. 2023. Readiness of nociceptor cell bodies to generate spontaneous activity results from background activity of diverse ion channels and high input resistance. Pain. 165:893–907. 10.1097/j.pain.0000000000003091 37862056 PMC10950548

[bib119] Tigerholm, J., M.E. Petersson, O. Obreja, E. Eberhardt, B. Namer, C. Weidner, A. Lampert, R.W. Carr, M. Schmelz, and E. Fransén. 2015. C-fiber recovery cycle supernormality depends on ion concentration and ion channel permeability. Biophys. J. 108:1057–1071. 10.1016/j.bpj.2014.12.034 25762318 PMC4816283

[bib120] Tigerholm, J., M.E. Petersson, O. Obreja, A. Lampert, R. Carr, M. Schmelz, and E. Fransén. 2014. Modeling activity-dependent changes of axonal spike conduction in primary afferent C-nociceptors. J. Neurophysiol. 111:1721–1735. 10.1152/jn.00777.2012 24371290 PMC4044369

[bib121] Tigerholm, J., A.H. Poulsen, O.K. Andersen, and C.D. Mørch. 2019. From perception threshold to ion channels-A computational study. Biophys. J. 117:281–295. 10.1016/j.bpj.2019.04.041 31255293 PMC6700668

[bib122] Tseng, A.S., W.S. Beane, J.M. Lemire, A. Masi, and M. Levin. 2010. Induction of vertebrate regeneration by a transient sodium current. J. Neurosci. 30:13192–13200. 10.1523/jneurosci.3315-10.2010 20881138 PMC2965411

[bib123] Usoskin, D., A. Furlan, S. Islam, H. Abdo, P. Lönnerberg, D. Lou, J. Hjerling-Leffler, J. Haeggström, O. Kharchenko, P.V. Kharchenko, . 2015. Unbiased classification of sensory neuron types by large-scale single-cell RNA sequencing. Nat. Neurosci. 18:145–153. 10.1038/nn.3881 25420068

[bib124] Vacher, H., D.P. Mohapatra, and J.S. Trimmer. 2008. Localization and targeting of voltage-dependent ion channels in mammalian central neurons. Physiol. Rev. 88:1407–1447. 10.1152/physrev.00002.2008 18923186 PMC2587220

[bib125] Vanoye, C.G., T.V. Abramova, J.M. DeKeyser, N.F. Ghabra, M.J. Oudin, C.B. Burge, I. Helbig, C.H. Thompson, and A.L. George Jr. 2024. Molecular and cellular context influences SCN8A variant function. JCI Insight. 9:e177530. 10.1172/jci.insight.177530 38771640 PMC11383174

[bib126] Vanoye, C.G., J.D. Kunic, G.R. Ehring, and A.L. George Jr. 2013. Mechanism of sodium channel NaV1.9 potentiation by G-protein signaling. J. Gen. Physiol. 141:193–202. 10.1085/jgp.201210919 23359282 PMC3557314

[bib127] Vasylyev, D.V., and S.G. Waxman. 2012. Membrane properties and electrogenesis in the distal axons of small dorsal root ganglion neurons in vitro. J. Neurophysiol. 108:729–740. 10.1152/jn.00091.2012 22572942

[bib128] Wang, D.W., K. Yazawa, A.L. George Jr., and P.B. Bennett. 1996. Characterization of human cardiac Na^+^ channel mutations in the congenital long QT syndrome. Proc. Natl. Acad. Sci. USA. 93:13200–13205. 10.1073/pnas.93.23.13200 8917568 PMC24070

[bib129] Wang, J., S.W. Ou, and Y.J. Wang. 2017. Distribution and function of voltage-gated sodium channels in the nervous system. Channels. 11:534–554. 10.1080/19336950.2017.1380758 28922053 PMC5786190

[bib130] Wang, T., Y. Wang, J. Shen, L. Wang, and L. Cao. 2022. Predicting spike features of Hodgkin-Huxley-type neurons with simple artificial neural network. Front. Comput. Neurosci. 15:800875. 10.3389/fncom.2021.800875 35197835 PMC8859780

[bib131] Whitaker, W.R., J.J. Clare, A.J. Powell, Y.H. Chen, R.L. Faull, and P.C. Emson. 2000. Distribution of voltage-gated sodium channel alpha-subunit and beta-subunit mRNAs in human hippocampal formation, cortex, and cerebellum. J. Comp. Neurol. 422:123–139. 10.1002/(sici)1096-9861(20000619)422:1<123:aid-cne8>3.0.co;2-x 10842222

[bib132] Wittmack, E.K., A.M. Rush, M.J. Craner, M. Goldfarb, S.G. Waxman, and S.D. Dib-Hajj. 2004. Fibroblast growth factor homologous factor 2B: Association with Nav1.6 and selective colocalization at nodes of Ranvier of dorsal root axons. J. Neurosci. 24:6765–6775. 10.1523/jneurosci.1628-04.2004 15282281 PMC6729706

[bib133] Yang, Y., Y. Wang, S. Li, Z. Xu, H. Li, L. Ma, J. Fan, D. Bu, B. Liu, Z. Fan, . 2004. Mutations in SCN9A, encoding a sodium channel alpha subunit, in patients with primary erythermalgia. J. Med. Genet. 41:171–174. 10.1136/jmg.2003.012153 14985375 PMC1735695

[bib134] Yin, K., G.J. Baillie, and I. Vetter. 2016. Neuronal cell lines as model dorsal root ganglion neurons: A transcriptomic comparison. Mol. Pain. 12:1744806916646111. 10.1177/1744806916646111 27130590 PMC4956150

[bib135] Yu, F.H., and W.A. Catterall. 2003. Overview of the voltage-gated sodium channel family. Genome Biol. 4:207. 10.1186/gb-2003-4-3-207 12620097 PMC153452

[bib136] Zhang, H., and A.S. Verkman. 2010. Aquaporin-1 tunes pain perception by interaction with Na(v)1.8 Na^+^ channels in dorsal root ganglion neurons. J. Biol. Chem. 285:5896–5906. 10.1074/jbc.M109.090233 20018876 PMC2820815

[bib137] Zhang, X., J.E. Hartung, and M.S. Gold. 2024. Persistent (Nav1.9) sodium currents in human dorsal root ganglion neurons. Pain. 10.1097/j.pain.0000000000003394 PMC11723807 39297710

[bib138] Zhang, X., B.T. Priest, I. Belfer, and M.S. Gold. 2017. Voltage-gated Na^+^ currents in human dorsal root ganglion neurons. Elife. 6:e23235. 10.7554/eLife.23235 28508747 PMC5433841

